# Catalytic Metal–Organic Framework Membranes: Advances in Electrocatalysis, Photocatalysis, and Thermocatalysis

**DOI:** 10.3390/nano16201306

**Published:** 2026-10-16

**Authors:** Jingzhe Deng, Fangye Zhang, Meng Wang, Yanwei Sun

**Affiliations:** 1 Faculty of Arts and Sciences, Beijing Normal University, Zhuhai 519087, China; 202311038797@mail.bnu.edu.cn (J.D.); 202311038574@mail.bnu.edu.cn (F.Z.); 202311039071@mail.bnu.edu.cn (M.W.); 2 Center for Advanced Materials Research, Advanced Institute of Natural Sciences, Beijing Normal University, Zhuhai 519087, China

**Keywords:** metal–organic framework (MOF) membranes, membrane catalysis, electrocatalysis, photocatalysis, thermocatalysis

## Abstract

Metal–organic framework (MOF) membranes combine the structural designability of MOFs with a continuous membrane architecture that integrates reactant enrichment, active-site accessibility, and product separation. This review surveys catalytic MOF membranes reported from 2016 to 2025, from fabrication strategies—classified by how catalytic function is integrated with the membrane (intrinsic framework active sites, guest species incorporation, and non-catalytic microenvironment coating)—to catalytic performance in electrocatalysis, photocatalysis, and thermocatalysis. In electrocatalysis, non-catalytic MOF coatings improve CO_2_ reduction and nitrogen reduction, whereas MOF-derived electrodes currently lead water-splitting benchmarks. In photocatalysis, MOF membranes have enabled CO_2_ reduction with near-unity HCOOH selectivity, pollutant degradation, Cr(VI) reduction, and H_2_ evolution stable for over 1000 h. In thermocatalysis, membrane demonstrations are so far confined to low- and medium-temperature antibacterial and oxidative desulfurization applications, whereas alkyne semihydrogenation and high-temperature operation of intact membranes remain largely unexplored. Across all three regimes, membrane geometry governs reactant enrichment, separation, and catalyst recoverability, yet inconsistent reporting limits cross-study comparison. Key challenges and future directions are discussed.

## 1. Introduction

Metal–Organic Frameworks (MOFs), also known as Porous Coordination Polymers (PCPs), are novel porous crystalline materials composed of organic ligands and metal centers linked through coordination bonds, exhibiting characteristics of both organic and inorganic materials [1,2,3]. Due to their high specific surface area, high porosity, tunable pore structure, and structural diversity [4], they are widely used in various fields such as gas storage and separation, catalysis, biosensing, drug delivery, and biomedical imaging [5,6].

MOF membranes retain the excellent properties of MOF materials while also exhibiting the selective permeability characteristics of membrane materials. MOF membranes are primarily classified into two categories: the first consists of pure MOF membranes prepared using a continuous growth method, which are composed of a polycrystalline MOF layer and a porous substrate; the second consists of mixed matrix membranes (MMMs), formed by mixing MOF particles with an organic matrix [7].

Early MOF membrane research originated from the pursuit of gas-separation membranes, benefiting from MOFs’ large specific surface area, tunable porosity, abundant metal sites and versatile chemical modifiability [8]. Nevertheless, early development faced two major bottlenecks: the controllable fabrication of defect-free continuous MOF layers and stable immobilization of MOF films on porous substrates [9]. In 2005, Fischer’s group reported the direct synthesis of polycrystalline MOF-5 thin films on pre-modified substrates, representing an important milestone for MOF thin-film fabrication, even though these early films exhibited no practical molecular-separation performance [9]. Subsequent efforts by Caro, Bein and other pioneers advanced strategies for MOF crystal growth on diverse substrate surfaces [10,11]. The year 2009 marked a pivotal turning point, when multiple groups realized the first intact, continuous MOF membranes dedicated to separation applications. Representative achievements include secondary-growth MOF membranes on porous α-alumina supports reported by Tsapatsis’ group, microwave-assisted synthesis of high-compatibility ZIF-8 membranes by Caro’s team, and the in situ solvothermal preparation of continuous MOF-5 membranes on α-alumina substrates by Lai and co-workers [12,13,14]. Later progress further included seed-induced hydrothermal growth for stable ZIF-8 membranes, manual-frictional ZnO deposition for improved mechanical stability, as well as liquid-phase epitaxy (LPE) and layer-by-layer (LBL) assembly to produce oriented ultra-thin MOF membranes with controllable defect density [1,15,16]. It should be emphasized that these fabrication breakthroughs developed originally for separation-oriented MOF membranes constitute essential structural prerequisites for catalytic MOF membranes. Catalytic MOF membranes similarly require continuous layers to avoid reactant bypass leakage, appropriate thickness to mitigate mass-transfer resistance, and sufficient mechanical and chemical robustness to survive catalytic operating environments.

In recent years, the focus of MOF membrane research has gradually expanded from separation to membrane catalysis and membrane reactors that integrate reaction with separation [17,18]. They exhibit high tunability and diversity in structural design and functionality, laying a solid foundation for catalytic applications. However, despite their vast potential, this field still faces numerous challenges. Under harsh industrial catalytic conditions, such as high temperatures and humidity, their structural stability and accessibility of active sites remain severely tested. Additionally, the large-scale, defect-free synthesis of high-performance MOF membranes and the precise balancing of catalytic activity, selectivity, and permeability remain major research challenges and focal points in this field.

**Figure 1:**
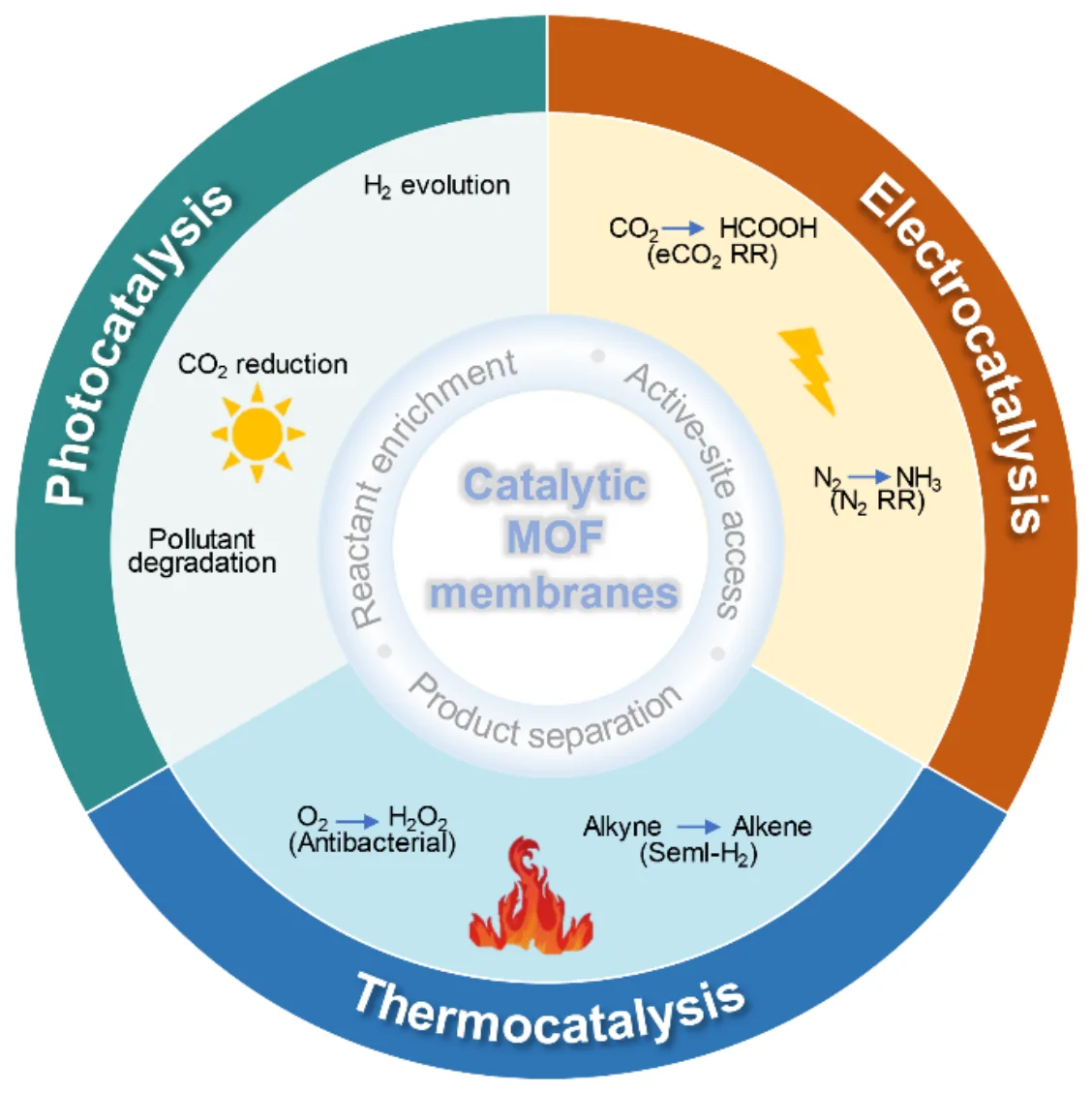
Schematic overview of catalytic MOF membranes, illustrating representative electrocatalytic, photocatalytic, and thermocatalytic reactions discussed in this review, unified by three shared membrane functions: reactant enrichment, active-site accessibility, and product separation.

Despite the rapid growth of MOF membrane research, the vast majority of existing reviews have focused predominantly on gas separation and liquid-phase filtration, leaving catalytic applications comparatively underrepresented in the literature [19,20]. While several reviews have addressed MOF-based catalysis in general, they typically treat MOFs as suspended powder catalysts rather than as structured membrane systems, thereby overlooking the unique advantages that membrane architecture confers—namely, continuous catalyst recovery, integrated reaction–separation operation, and the ability to exploit transmembrane concentration gradients to drive selectivity. Equally, reviews of membrane catalysis in the broader sense seldom address MOFs specifically, missing the distinctive structural tunability and active-site designability that set MOF membranes apart from conventional inorganic or polymeric catalytic membranes. This gap between the MOF membrane community and the membrane catalysis community means that the synergistic potential of combining both fields has yet to be systematically articulated. Furthermore, the past decade (2016–2025) has witnessed transformative advances in this intersection—from the first proof-of-concept photocatalytic MOF membranes to single-atom-loaded MOF membrane reactors capable of near-unity selectivity—developments that have not been captured in any single comprehensive account. A dedicated, up-to-date review that bridges fabrication methodology, mechanistic understanding, and application performance across the three principal catalytic modalities (electrocatalysis, photocatalysis, and thermocatalysis) is therefore both timely and necessary (Figure 1).

This review covers catalytic membranes constructed from both transition-metal-based MOFs and emerging lanthanide-based (Ln-) MOF families. While transition-metal MOF membranes dominate current literature reports, Ln-MOF membranes represent a promising, fast-developing subclass with unique 4f-orbital-derived physicochemical properties [21], even though many studies are still confined to powder-state materials and membrane-scale demonstrations remain limited.

**Figure 2:**
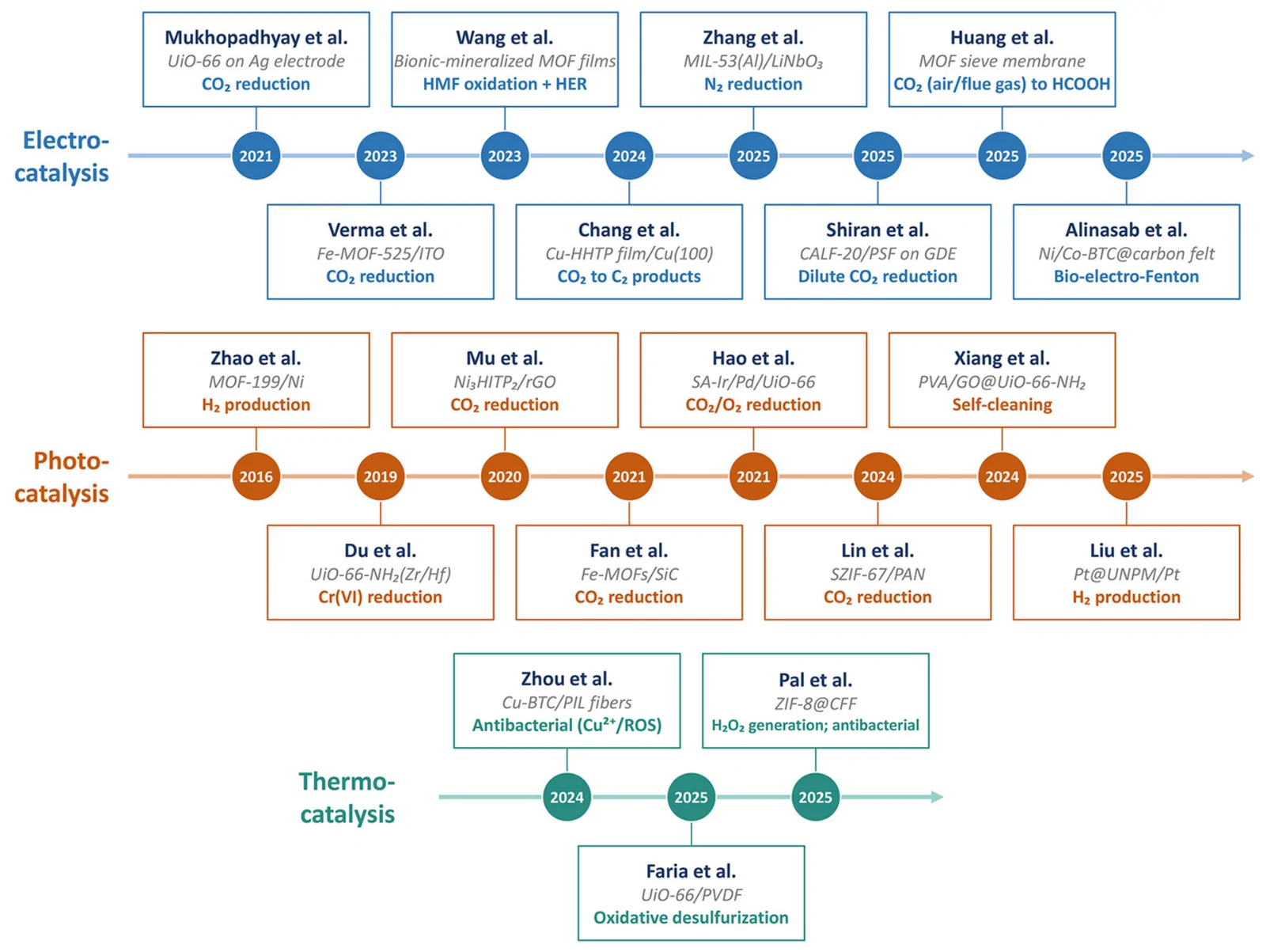
Timeline of representative milestones in the development of catalytic MOF membranes (2016–2025), organized by catalytic regime (electrocatalysis, photocatalysis, thermocatalysis). Each entry lists the first author, publication year, the MOF membrane system, and the catalytic reaction studied, based on the works reviewed in Section 3. The entries correspond to the following references. Electrocatalysis: Mukhopadhyay et al. [22], Verma et al. [23], Wang et al. [24], Chang et al. [25], Zhang et al. [26], Shiran et al. [27], Huang et al. [28], Alinasab et al. [29]; photocatalysis: Zhao et al. [30], Du et al. [31], Mu et al. [32], Fan et al. [33], Hao et al. [34], Lin et al. [35], Xiang et al. [36], Liu et al. [37]; thermocatalysis: Zhou et al. [38], Faria et al. [39], Pal et al. [40].

Therefore, this paper aims to systematically review the research progress on MOF membranes in the field of catalysis from 2016 to 2025, with particular attention to the structure–activity relationships that govern catalytic performance in membrane form (Figure 2). The scope of this review encompasses both pure-phase MOF membranes and MOF-based mixed matrix membranes (MMMs) that function as active catalytic components, spanning three principal catalytic regimes: electrocatalysis (including water splitting and electrochemical CO_2_ reduction), photocatalysis (including solar-driven CO_2_ reduction, pollutant degradation, heavy metal removal, and photocatalytic H_2_ evolution), and thermocatalysis (including low- to mid-temperature selective reactions and antimicrobial applications). MOF membranes used purely for physical separation without a catalytic function, as well as MOF-derived materials in which the original framework is fully decomposed prior to use, fall outside the scope of this review. We begin with the fabrication strategies used to construct catalytically functional MOF membranes (Section 2), then systematically examine representative works and mechanistic insights across the three catalytic regimes (Section 3), and conclude with a forward-looking perspective on key challenges and emerging directions (Section 4).

Across these three catalytic regimes, the case studies surveyed in this review reveal a common design logic that recurs regardless of the driving energy source: the catalytic function of a MOF membrane is realized through one of three integration modes. In the first, intrinsic framework active sites, the MOF’s own metal nodes or coordinatively unsaturated defect sites directly catalyze the reaction, as in the Ni-, Cu-, and Zr-based frameworks used below for electrocatalytic hydrogen evolution, photocatalytic CO_2_ reduction, and catalytic oxidative desulfurization. In the second, guest species incorporation, metal nanoparticles, single atoms, or molecular catalysts are hosted within the MOF pores while the framework itself plays a secondary, structure-directing role, as in single-atom-loaded MOF membranes and MOF-templated phosphide electrocatalysts. In the third, non-catalytic microenvironment coating, an electrically or catalytically inert MOF layer is grown directly onto an independent catalyst or electrode purely to engineer the local reaction environment, as first demonstrated for electrocatalytic CO_2_ and N_2_ reduction on non-catalytic UiO-66 and MIL-53(Al) membranes. This classification, developed in detail in Section 2, provides the organizing thread that connects fabrication strategy (Section 2) to catalytic performance (Section 3) throughout the review.

## 2. Fabrication Methods for Catalytic MOF Membranes

Underlying the specific fabrication protocols surveyed below, it is useful to first distinguish the fabrication routes according to how the catalytic function is integrated with the membrane, since this determines which preparation route is appropriate. Three integration modes recur throughout the literature: (i) the MOF framework itself supplies the catalytic sites (e.g., unsaturated metal nodes or defect sites), so fabrication need only preserve framework integrity; (ii) a guest catalytic species—metal nanoparticles, single atoms, or a second semiconductor—is incorporated within or alongside the MOF, so fabrication must additionally control the location and dispersion of that guest; and (iii) a non-catalytic MOF layer is coated onto an independent catalyst or electrode purely to engineer the local reaction microenvironment, so fabrication must prioritize conformal, defect-free coverage over the underlying catalyst. The four preparation techniques described next—in situ growth, solution-based polymer processing, vacuum filtration, and powder-based assembly—are the practical means by which each of these three integration modes is realized.

Various strategies have been developed for the preparation of MOF membranes with catalytic properties. Depending on the substrate type, target application, and structural design, these methods can be classified into four main categories: in situ growth, solution-based polymer processing, vacuum filtration, and powder-based assembly. Each approach is tailored to specific applications and features distinct preparation protocols.

### 2.1. In Situ Growth

In situ growth is widely used to fabricate continuous, catalytically active MOF films, where MOF crystals are directly synthesized and grown on the surface of a substrate, support, or an existing catalyst. For instance, Zhang et al. directly grew an ultrathin MIL-53(Al) film onto a LiNbO_3_ perovskite oxide electrocatalyst, exploiting the strong affinity between the MOF’s Al–O–Al bridging-oxygen sites and N_2_ to enrich the reactant at the catalyst interface for electrocatalytic nitrogen reduction [26].

This method creates tight interfacial contact between the MOF layer and the underlying substrate or catalyst, which lowers interfacial charge-transfer resistance for electro- and photocatalytic systems. It supports conformal coating over substrates with complex geometries and enables direct deposition of non-catalytic MOF microenvironment coatings onto pre-fabricated electrodes. Strong material adhesion obtained via in situ growth also helps mitigate MOF exfoliation during liquid-phase catalytic operation. Nevertheless, nucleation behavior is highly sensitive to substrate surface chemistry, and surface pre-modification is generally required to trigger heterogeneous MOF crystal formation [41]. Most solvothermal in situ growth relies on high-temperature, high-pressure autoclave conditions, which creates barriers toward large-scale continuous manufacturing. Independent fine-tuning of membrane thickness, defect density and guest-species loading remains experimentally challenging, and undesired bulk MOF precipitation in the mother liquor often competes against heterogeneous surface nucleation. This technique is therefore best suited for electrocatalytic micro-environment modification of existing electrodes, preparation of tightly anchored thin catalytic MOF films for photocatalysis, and other operating environments that demand robust MOF-substrate adhesion under dynamic flow conditions.

### 2.2. Solution-Based Polymer Processing

Solution-based polymer processing techniques, including solution casting and electrospinning, are used to embed catalytically active MOF particles within a flexible polymer matrix. Fan et al. first electrospun polyvinyl alcohol-ethylene-polyethylene (PVA-co-PE) nanofibers, then grew Fe-MOFs/SiC in situ on the fiber surface, yielding a composite membrane with excellent photocatalytic CO_2_-reduction activity under visible light [33]. Using a similar electrospinning route, Lin et al. grew ZIF-67 in situ on electrospun polyacrylonitrile (PAN) nanofiber membranes, followed by a thermal stabilization treatment, to obtain SZIF-67/PAN membranes with enhanced visible-light responsiveness and photothermal conversion, which likewise exhibited excellent photocatalytic activity for CO_2_ reduction [35,42].

This family of techniques delivers freestanding, mechanically robust mixed-matrix membranes compatible with roll-to-roll processing, offering practical prospects for real-world water-treatment catalytic filtration. The polymer host matrix can shield chemically vulnerable MOF crystallites against hydrolysis and corrosive aqueous surroundings, and MOF filler loading can be readily adjusted by modifying feedstock composition. Yet practical performance is constrained by material compatibility limits. Elevated MOF loading frequently triggers particle aggregation and interior membrane defects, while low filler content results in insufficient density of catalytic active sites [43]. Polymer chains may fully encapsulate MOF grains, reducing the number of exposed active sites and the apparent specific surface area of the composite. Most commercial polymer matrices cannot withstand temperatures above 200 °C, which restricts deployment in high-temperature thermocatalysis [44]. Poor interfacial compatibility between MOF fillers and polymer matrices can further generate non-selective interstitial gaps that cause reactant bypass. Solution-based polymer processing finds its greatest utility in mild-aqueous photocatalytic applications including water pollutant degradation, heavy-metal remediation and photocatalytic CO_2_ reduction, as well as flow-through catalytic filtration systems and portable flexible catalytic membrane devices.

### 2.3. Vacuum Filtration

Vacuum filtration is well suited for assembling composite films of two-dimensional conductive MOFs with carbon materials, offering a simple procedure and good film uniformity. Mu et al. exploited this method to co-deposit the two-dimensional conductive MOF Ni_3_HITP_2_ with reduced graphene oxide (rGO): negatively charged rGO nanosheets and positively charged Ni_3_HITP_2_ particles were combined via electrostatic interaction, and the resulting suspension was vacuum-filtered, freeze-dried, and cut into pieces to yield a Ni_3_HITP_2_/rGO heterostructure film. The intimate 2D/2D interface enhances electron density at the catalytic Ni sites and accelerates photocatalytic CO_2_ reduction [32].

Assembly proceeds under mild ambient conditions without high-temperature treatment and enables the construction of tight 2D/2D heterointerfaces between conductive MOFs and carbon nanomaterials such as reduced graphene oxide and carbon nanotubes. Film thickness can be conveniently controlled by adjusting suspension concentration and filtered volume, and elaborate substrate pre-treatment is not mandatory. The mechanical integrity of as-prepared films largely depends on inter-particle van der Waals attraction; membranes may disintegrate upon long-term liquid-phase stirring or cross-flow filtration. The approach works primarily for nanosheet-shaped or small-sized MOF building blocks, while large MOF crystallites produce heterogeneous, leak-prone films. Fabricating large-area defect-free membranes remains difficult, and thermal tolerance is limited. Vacuum filtration is most appropriate for lab-scale proof-of-concept electro- and photocatalytic thin-film devices, conductive MOF-carbon composite catalysts for small-volume batch reactions, and fundamental mechanistic investigations on 2D-MOF heterostructure catalysis, rather than industrial continuous-flow operations [45].

### 2.4. Powder-Based Assembly

Powder-based assembly is a non-in situ strategy in which catalytically functionalized MOF particles are pre-synthesized and subsequently assembled into a membrane via deposition or coating onto a porous support. Hao et al. incorporated single-atom iridium and palladium centers into defect-engineered A-aUiO particles by impregnation and annealing, then deposited the resulting SA/A-aUiO particles layer-by-layer onto a porous polytetrafluoroethylene (PTFE) membrane, yielding a gas-permeable SA/MOF membrane that catalyzes the photoreduction of CO_2_ to HCOOH and O_2_ to H_2_O_2_ with near-unity selectivity [34].

Decoupling MOF powder synthesis from membrane fabrication allows full material characterization and catalytic pre-optimization of MOF powders, including single-atom-doped and defect-engineered variants, before membrane formation. Porous supports including ceramics, PTFE and metal meshes can be selected flexibly for target scenarios, and layer-by-layer deposition grants precise control over membrane thickness and hierarchical pore architecture. This workflow also avoids destructive post-synthetic chemical treatments after the membrane has already been formed. Grain-boundary gaps among pre-synthesized MOF particles commonly create pinhole-type non-selective defects that permit reactant bypass. Physical adhesion between the MOF powder layer and underlying support is relatively weak, raising risks of particle shedding under flow conditions. Multi-step layer-by-layer protocols are time-intensive and challenging to scale up. Powder-based assembly is therefore favored for gas–solid interfacial photocatalysis using pre-modified single-atom MOF catalysts, performance validation of well-characterized complex MOF catalytic powders under membrane configuration, and fundamental evaluation of guest-species-incorporated MOF membrane systems.

### 2.5. Emerging Auxiliary Fabrication Strategies

Beyond the four mainstream routes mentioned above, several emerging techniques are gradually being adopted for catalytic MOF membrane construction, although most remain at laboratory scale. Electrophoretic deposition drives charged MOF nanoparticles to migrate and deposit onto conductive substrates under an external electric field, enabling rapid preparation of thin uniform MOF films on complex conductive electrodes for electrocatalysis [46,47]. However, controlling grain intergrowth and eliminating pinhole defects still represent major challenges. Atomic/molecular layer deposition (ALD/MLD) offers a solvent-free alternative in which MOF thin films are grown directly from gaseous metal and organic-linker precursors with nanometre-level thickness control and conformal coverage; however, this vapor-phase route increases equipment cost and process complexity [48]. Interfacial gelation realizes MOF membrane formation at liquid–liquid or liquid-air interfaces, yielding large-area freestanding MOF nanofilms; yet mechanical fragility severely restricts its practical catalytic deployment.

For all these emerging methods, further investigation is still required to balance catalytic activity, defect density, mechanical robustness and manufacturing cost before real catalytic application. The selection of fabrication strategy should not only match the chemical nature of the target MOF material, but also fully consider the intended catalytic integration mode (intrinsic active sites, guest incorporation, non-catalytic micro-environment coating) and practical operating conditions of the final catalytic membrane reactor [49,50,51,52,53].

## 3. MOF Membranes in Different Catalytic Systems

Two classes of materials appear in this section and should not be benchmarked on the same footing. Class I membranes retain the crystalline framework and organic linkers during operation (e.g., MIL-53(Al)/LiNbO_3_, UiO-66/Ag, Ni_3_HITP_2_/rGO); their performance can legitimately be attributed to framework features such as pore confinement, open metal sites, or linker functionality. Class II membranes use the MOF only as a template or precursor, and the framework is partially or fully converted before use (e.g., Co_2_P/CoP@C, MOF@CoP); here, the MOF membrane contributes geometry and precursor chemistry, but catalysis occurs on the derived phase. We therefore (i) discuss Class II examples only as benchmarks that define the performance that intact-framework membranes must match, and (ii) recommend that studies report post-reaction XRD, FTIR/Raman, XPS, and metal leaching (ICP) to establish which class a material belongs to.

### 3.1. Electrocatalytic Applications

Electrocatalytic reaction–product separation integration centers on the coordinated design of reactors and separation modules as its core objective. Through structural regulation and property optimization of functional materials, this strategy realizes efficient in situ separation and purification of target products, thereby boosting the overall efficiency and operational safety of the reaction system.

#### 3.1.1. Electrocatalytic Water Splitting

Electrocatalytic water splitting into H_2_ and O_2_ is a cornerstone reaction for green hydrogen production, yet practical electrolyzers remain constrained by the sluggish kinetics of the oxygen evolution reaction (OER) and the cost of noble-metal catalysts. Cui et al. fabricated a MOF@CoP hybrid bifunctional electrocatalytic nanofilm directly on a carbon fibrous membrane via in situ growth followed by controlled phosphorization; because the MOF component is retained within the final hybrid rather than fully decomposed, the continuous nanofilm architecture maintains intimate contact between the MOF-derived CoP phase and the conductive carbon-fiber support, improving charge transfer while the membrane’s porosity preserves facile gas and electrolyte transport for overall water splitting [54]. A related but mechanistically distinct strategy uses the MOF membrane not as the final catalyst but as a structure-directing template: Zhang et al. first grew an oxygen-coordinated MOF into a continuous membrane on a graphite substrate via cathodic electrodeposition, then fully converted this MOF membrane into a supported Co_2_P/CoP@C heterostructure through phosphorization; the resulting electrode required only 146 and 365 mV overpotentials to reach 100 mA·cm^−2^ for the HER and OER, respectively, with the high performance attributed to the conductive pathway and Co_2_P/CoP interface established during the templating step. Because the framework is fully decomposed in this second case, it more strictly represents a MOF-membrane-templated catalyst than an intact catalytic MOF membrane, but it is included here to illustrate how the membrane geometry established during MOF growth can be inherited by the derived catalyst even after the organic framework itself is removed [55].

Beyond bifunctional water-splitting nanofilms, MOF membranes have also been engineered to promote HER selectively through protic-salt engineering of the metal precursor. Mishra et al. fabricated NiO-based MOF membranes by mixing NiO with a protic ionic liquid—either dimethylethylamine (dmea) or diethylmethylamine (dema)—followed by stepwise thermal curing, yielding a crystalline Pbca-symmetry framework with a distorted diamondoid topology. The dmea-templated membrane outperformed its dema-templated counterpart, exhibiting a Tafel slope as low as 30 mV·dec^−1^ consistent with a single-electron-transfer HER pathway, a catalytic current density of 590 mA·cm^−2^, a turnover frequency of 1.8408 × 10^18^ s^−1^, and stable operation for 168 h (7 days) at 1 A·cm^−2^ in neutral electrolyte; the improvement was attributed to a smoother, more homogeneous crystallite morphology and stronger Ni–N coordination relative to the dema-templated membrane, illustrating how the choice of structure-directing protic salt can be used to tune both the mechanical and electrocatalytic properties of a MOF membrane electrode [56].

In the realm of scalable fabrication, Wang et al. integrated metal corrosion with biomimetic mineralization to prepare 16 types of MOF films—encompassing carboxylate-, N-heterocycle-, phenolate- and phosphonate-based frameworks—on seven metals under mild conditions [24]. These films can be patterned over length scales from micrometers to meters by techniques such as inkjet printing. In a large-area electrolyzer (64 cm^2^), the MOF films catalyzed the oxidation of 5-hydroxymethylfurfural coupled with hydrogen evolution, reaching a current of 3.0 A at 1.63 V. By replacing the sluggish OER with organic oxidation, this work shows how MOF films can simultaneously enable value-added chemical synthesis and hydrogen production at scale.

#### 3.1.2. Electrocatalytic CO_2_ Reduction

The electrochemical reduction of CO_2_ (eCO_2_RR) to value-added fuels and chemicals represents a critical strategy for closing the anthropogenic carbon cycle. A central challenge in eCO_2_RR is the low local concentration of CO_2_ at the electrode surface in aqueous media (~0.03 M at neutral pH), which leads to sluggish reaction kinetics and poor product selectivity. MOF membranes have recently emerged as a powerful microenvironment-engineering strategy to address this bottleneck. By assembling a porous MOF layer directly onto a heterogeneous electrocatalyst surface, the membrane modulates mass transport, tunes the local chemical environment at active sites, and can stabilize reaction intermediates through secondary coordination sphere interactions—all without contributing catalytic activity itself.

**Figure 3:**
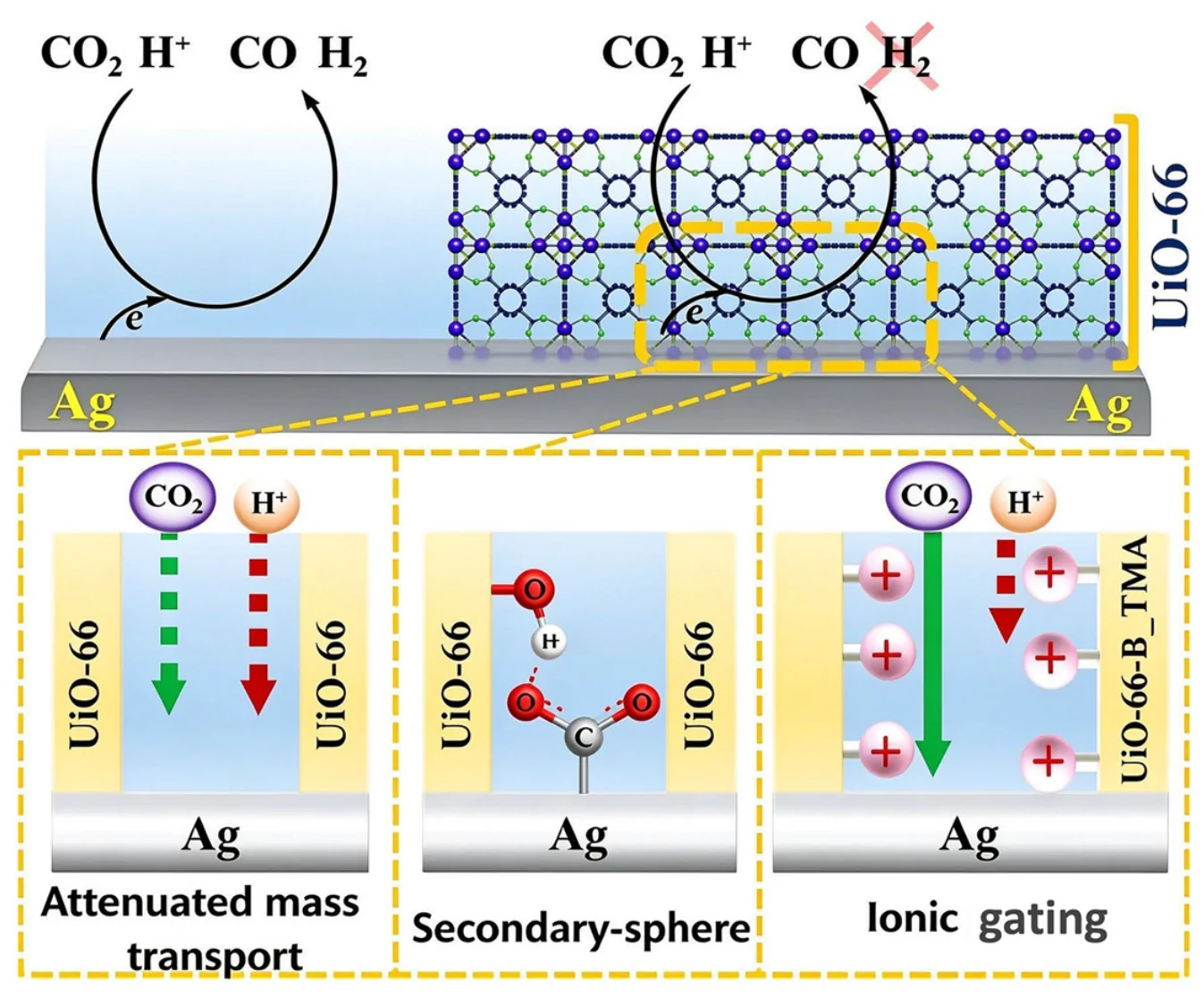
Illustration of the structure of a UiO-66 MOF-membrane enhancing CO_2_-to-CO catalysis when grown over Ag. Adapted with permission from [22]. Copyright 2021, Wiley.

Mukhopadhyay et al. first demonstrated the concept of a non-catalytic MOF acting as a functional membrane to control heterogeneous electrocatalysis. They assembled an electrically insulating UiO-66 MOF layer directly onto a silver (Ag) CO_2_RR electrocatalyst. Despite carrying no intrinsic catalytic function, the UiO-66 membrane markedly enhanced both activity and CO selectivity by regulating the delivery of CO_2_ and protons toward the Ag active sites through physical and electrostatic mechanisms (Figure 3). Post-synthetic modification of the MOF with a positively charged trimethylammonium ligand (TMA) introduced an electrostatic field that further steered CO_2_ toward the electrode while repelling competing anions. The MOF also stabilized key reaction intermediates through secondary coordination-sphere interactions within the pore channels, offering a previously unreported degree of molecular-level control over a heterogeneous catalytic surface. This proof-of-concept study established the paradigm of “membrane-as-microenvironment-engineer”, demonstrating that the chemical modularity of MOFs could be leveraged as a new dimension for improving eCO_2_RR selectivity [22].

Building upon this strategy of regulating mass transfer with a non-catalytic membrane, subsequent studies have extended the target feed from pure CO_2_ to dilute CO_2_ sources such as flue gas and even ambient air. Shiran et al. fabricated a selective mixed-matrix layer on the surface of a gas diffusion electrode by loading CO_2_-philic CALF-20 MOF particles into a polymer matrix, and systematically compared three matrices—Nafion, polysulfone (PSF) and styrene–ethylene–butylene–styrene (SEBS)—in terms of CO_2_/N_2_ selectivity and permeation flux [27]. The CALF-20/PSF layer exhibited a CO_2_/N_2_ selectivity of approximately 40; even with a feed containing only 10 vol% CO_2_, it delivered a CO partial current density about three times that of an unmodified electrode (~42.7 mA·cm^−2^) while maintaining a CO Faradaic efficiency of ~70% for over 20 h in a membrane-electrode assembly. Under even more dilute conditions, Huang et al. embedded a self-supporting MOF-based molecular-sieve membrane directly into an electrolyzer, assembled together with a conductive diffusion layer and a Bi nanoparticle catalyst layer [28]. The membrane enriched CO_2_ from 15% to 82.5% in flue gas and from 0.04% to 2.05% in air while rejecting interfering SO_2_, NO and O_2_, so that the enriched CO_2_ could be reduced directly to formic acid under acidic conditions; with flue gas as the feed, a total current of up to 9000 mA was achieved with a formic acid Faradaic efficiency approaching 100%. These two studies show that non-catalytic MOF membranes have evolved from regulating local mass transfer in pure CO_2_ systems to serving as an inlet layer that combines gas purification, in situ CO_2_ enrichment and impurity rejection, offering a route to bypass a separate, energy-intensive carbon-capture step.

A complementary route toward eCO_2_RR-active MOF membranes uses meniscus-guided solution shearing to grow large-area, oriented thin films directly on a conductive substrate. Verma et al. produced continuous MOF-525 thin films on indium tin oxide (ITO) via solution shearing, then post-synthetically metalated approximately 20% of the porphyrin linkers with Fe^3+^ to obtain an electrocatalytically active Fe-MOF-525 film while preserving the parent crystal structure and film morphology. In CO_2_-saturated organic electrolyte, the Fe-metalated film substantially outperformed both bare ITO and the non-metalated MOF-525 film for electrocatalytic CO_2_ reduction to CO, whereas the unmetalated parent film showed negligible intrinsic activity, confirming that in this system the MOF thin film itself must carry a catalytically competent metal site rather than acting purely as a microenvironment modulator—an instructive contrast to the non-catalytic UiO-66 membrane strategy described above [23].

MOF films that carry catalytic sites can also use framework cavities to confine active metal species and thereby stabilize active oxidation states under electroreduction conditions. Chang et al. grew a two-dimensional conductive Cu(II)-HHTP film on Cu(100) foil by in situ electrochemical assembly; a subsequent pre-reduction step converted part of the Cu(II) to Cu(I), generating highly dispersed Cu_2_O clusters within cavities formed by localized rupture of the framework [25]. Confined in these cavities and stabilized by the HHTP ligands, the Cu(I) clusters resisted further reduction to Cu^0^, creating a dual-site microenvironment of coexisting Cu(I)(111) and Cu^0^(100). This configuration delivered a C_2_ product selectivity of 72.6% at current densities above 300 mA·cm^−2^ with stable operation for more than 50 h; in situ spectroscopy and theoretical calculations indicated that the confined Cu(I) sites lowered the energy of the *OCCOH coupling pathway while the underlying Cu^0^ sites increased *CO coverage.

#### 3.1.3. Electrocatalytic Nitrogen Reduction

The electrochemical nitrogen reduction reaction (NRR) under ambient conditions offers a promising low-energy pathway for sustainable ammonia synthesis as an alternative to the energy-intensive Haber–Bosch process. However, the extremely low solubility of N_2_ in aqueous electrolyte and the dominance of the competing hydrogen evolution reaction (HER) severely limit both NH_3_ yield rate and Faradaic efficiency (FE). MOF membranes have recently been shown to address both obstacles simultaneously—by preferentially adsorbing N_2_ at the catalyst interface and simultaneously repelling water molecules—thereby enriching N_2_ locally while suppressing proton supply to the competing HER.

Zhang et al. demonstrated this dual-function mechanism in a composite system (LN@MIL-X) fabricated by coating an ultrathin MIL-53(Al) MOF membrane onto a LiNbO_3_ perovskite oxide electrocatalyst. MIL-53(Al) was selected for its outstanding N_2_ affinity arising from the strong interaction between its Al–O–Al bridging-oxygen sites and the N_2_ molecule, as established by grand canonical Monte Carlo (GCMC) simulations and corroborated by in situ diffuse reflectance infrared Fourier transform spectroscopy (DRIFTS). The MIL-53(Al) coating thickness was tuned by varying the MOF-to-substrate mass ratio (denoted LN@MIL-0.2, LN@MIL-0.4, and LN@MIL-0.6). The optimized LN@MIL-0.4 composite exhibited a maximum NH_3_ yield rate of 45.25 μg·h^−1^·mg^−1^·cat. with a significantly enhanced Faradaic efficiency compared to pristine LiNbO_3_. The improvement was attributed to the MOF membrane’s selective N_2_ recognition and concentration at the perovskite surface while simultaneously acting as a hydrophobic barrier to repel water molecules, thereby suppressing the competing HER and improving the N_2_ utilization efficiency. Taken together, the eCO_2_RR and NRR studies described above reveal a unifying design logic: the MOF membrane need not be a catalyst itself—its value lies in creating a privileged chemical environment at the electrode–electrolyte interface, selectively concentrating the desired reactant, repelling competing species, and stabilizing productive intermediates. This microenvironment engineering approach is broadly transferable and opens a new dimension of rational design for electrochemical small-molecule activation [26].

#### 3.1.4. Electrocatalytic Pollutant Degradation

Beyond energy-conversion reactions, electrocatalytic MOF membranes have also been developed to degrade organic pollutants and chemical contaminants directly within the membrane, combining separation with in situ oxidative detoxification.

Zhao et al. integrated a MOF-808 catalytic interlayer into a bipolar membrane, sandwiched between a sulfonated cation-exchange layer and an imidazolium-functionalized anion-exchange layer; under an applied electric field, the MOF-808 interlayer catalyzed water dissociation with 93.3% efficiency within 1 h, generating hydroxide in situ that degraded the nerve-agent simulant DMNP to 95% conversion within 128 min without external alkali addition, while retaining most of its degradation efficiency after 20 cycles [57].

Ye et al. constructed a Cu-TCPP MOF/MXene/AgNWs ternary electrocatalytic ultrafiltration membrane by blending the conductive filler network into a polyethersulfone matrix; under a modest 5 V bias, the membrane achieved over 90% degradation of several dyes (methylene blue, sunset yellow, crystal violet, malachite green) within 5 h, alongside high ultrafiltration rejection (>98% for Congo red) and an almost three-fold flux enhancement over the unmodified polymer membrane, with hydroxyl radicals identified as the dominant reactive species [58].

Zhang et al. took a resource-recovery approach, electrochemically decomplexing heavy-metal–EDTA chelates from simulated electroplating wastewater and converting the recovered Cu, Pb, and Zn directly into a mixed-metal CuPbZn-BTC electrocatalytic membrane; the resulting multi-metal active sites generated abundant oxygen vacancies and Lewis acid sites, enabling tunable radical (•OH, •O^2−^) and non-radical (O_2_) degradation pathways that removed 92–99% of antibiotics without added chemical oxidants, illustrating how electrocatalytic MOF membranes can couple heavy-metal remediation with downstream antibiotic degradation [59].

Alinasab et al. cathodically deposited a Ni/Co-BTC MOF onto carbon felt and graphite plate to build a bio-electro-Fenton system in which exoelectrogen-driven H_2_O_2_ generation is coupled with MOF-catalyzed oxygen reduction [29]; the Ni/Co-BTC@carbon-felt cathode delivered a maximum power density of 133.6 mW·m^−2^ while removing 98.0%, 84.0% and 41.0% of bisphenol S at feed concentrations of 1, 5 and 10 mg·L^−1^ within 24 h, illustrating how MOF-modified bio-electrodes can pair pollutant destruction with concurrent electricity generation.

Ahmad et al. further advanced this energy-self-sufficient route by decorating a laser-induced graphene cathode with MIL-88B(Fe) for a microbial-fuel-cell-driven Fenton process [60]. The Fe-MOF cathode raised in situ H_2_O_2_ generation to 3.22 mg·L^−1^ (1.2- and 1.5-fold higher than bare laser-induced graphene and carbon felt) and achieved 95.3% ciprofloxacin removal within 180 min with 42.4% total organic carbon abatement and a power density of 250.2 mW·m^−2^; it still removed 90.7% of ciprofloxacin in secondary-treated real wastewater with minimal iron leaching, providing the type of real-matrix validation that most single-contaminant laboratory studies lack.

Across the electrocatalytic membranes compared in Table 1, only two report benchmarkable electrochemical metrics under standard protocols—overpotential at a fixed current density (Zhang et al.’s Co_2_P/CoP@C, 146/365 mV at 100 mA·cm^−2^) and Tafel slope with long-duration galvanostatic testing (Mishra et al.’s NiO/dmea, 30 mV·dec^−1^, stable at 1 A·cm^−2^ for 168 h). Tellingly, both of these best-performing entries depart from the strict definition of an intact catalytic MOF membrane: one fully sacrifices the framework during phosphorization, and the other relies on a protic-salt-cured, structurally MOF-like but not fully crystalline network. This suggests that, for demanding high-current-density reactions such as HER/OER, MOF-derived or MOF-templated strategies currently outperform intact-framework MOF membrane catalysis—an uncomfortable but important observation for a review centered on “catalytic MOF membranes.” By contrast, the non-catalytic microenvironment-coating strategy (UiO-66/Ag, MIL-53(Al)/LiNbO_3_) is mechanistically the most elegant application of the membrane concept, yet neither study reports an absolute Faradaic efficiency or current density that can be benchmarked against non-MOF electrodes, leaving its practical competitiveness unproven. The pollutant-degradation subset is strong on percentage removal (92–99%) but weak on turnover-based kinetics and real-water validation, with most tests run in clean, single-contaminant model systems. Taken together, the central limitation of this subfield is not performance but comparability: no two studies report stability, intrinsic activity, and Faradaic efficiency using a common protocol, so any ranking across systems remains qualitative rather than quantitative.

Ex situ PXRD and XPS are widely used to verify MOF crystallinity and surface valence states, and sample preparation is straightforward. However, conventional ex situ characterization alone is insufficient to follow the structural evolution of MOFs under applied electrochemical bias [61], so these techniques are best suited to preliminary material screening rather than tracking catalyst evolution under real operating conditions. Operando electrochemical Raman spectroscopy and synchrotron-based X-ray absorption spectroscopy (XAS/XAFS) are among the most informative approaches for probing such dynamics and for verifying whether the framework, particularly at the MOF surface, is retained during operation [61]; operando UV–vis, Raman, infrared and X-ray absorption methods have likewise been applied to electrocatalytic MOFs and COFs [62]. Their drawbacks are access and experimental complexity: synchrotron beam time is limited and dedicated electrochemical cells add further requirements, although laboratory-based XAS setups with resolution comparable to synchrotron systems have been reported [61]. These tools have therefore been applied mainly in fundamental mechanistic studies rather than routine screening. Extending them to intact MOF membranes in working cells would allow the intact-versus-derived question raised in Section 3 to be settled directly.

Lack of unified electrochemical testing standards hinders cross-study comparison, as performance is reported using inconsistent mass- or area-normalized metrics. It remains difficult to separate the catalytic contributions of intact MOF frameworks versus reconstructed derived phases, calling for more operando characterizations to verify MOF structural stability under high-current electrolysis. While non-catalytic MOF coating improves selectivity in lab-scale cells, high-current-density performance and long-term galvanostatic stability (>100 h) under practical cell conditions are rarely evaluated. Pollutant-degradation tests are mostly limited to synthetic wastewater; testing with real, multi-ion-containing water matrices is needed. Scalable fabrication of large-area, defect-free MOF membranes remains an open challenge [63], and this limitation is likely to apply equally to electrocatalytic MOF-membrane electrodes.

### 3.2. Photocatalytic Applications

MOFs exhibit distinctive properties for photocatalytic applications, including tunable light-harvesting ability, promoted electron–hole separation, and homogeneously dispersed catalytic active sites. However, highly dispersed powdered MOF nanoparticle materials are difficult to separate from suspension systems, leading to poor catalyst recovery and making recycling challenging. In contrast, continuous MOF membranes can maintain MOF activity and retain the advantages of MOF powders whilst facilitating separation from products, thereby enhancing the catalyst’s recyclability. The following section will highlight the advantages of MOF membranes in the field of photocatalysis by introducing their applications in CO_2_ reduction, pollutant degradation, heavy metal reduction and removal, and efficient hydrogen production. Beyond widely investigated transition-metal-based MOF membranes, lanthanide-based (Ln-) MOF membranes represent an emerging subclass. Ln-MOFs are distinguished by their 4f electronic configurations, higher and more variable coordination numbers, and characteristic luminescence, and have been explored in catalytic and photocatalytic applications [64,65]; yet membrane-form implementations remain far less mature than their powder counterparts and have so far been demonstrated mainly for luminescent sensing and pollutant removal [66,67,68].

#### 3.2.1. CO_2_ Reduction

MOFs possess advantages such as an extremely high specific surface area, tunable ordered porous structures, high CO_2_ adsorption capacity, good compatibility with polymers, and a high degree of design flexibility. By concentrating or enriching CO_2_ molecules near active sites, they offer specific advantages in the photocatalytic conversion of CO_2_ into high-value chemicals. Consequently, the synthesis of MOF membrane materials—which utilize the rigid porous structure of the membrane to facilitate the easy separation of the photocatalyst from the reaction system and enhance the long-term stability of the catalyst—has emerged as an excellent approach to enhancing the photocatalytic CO_2_ reduction activity [69].

**Figure 4:**
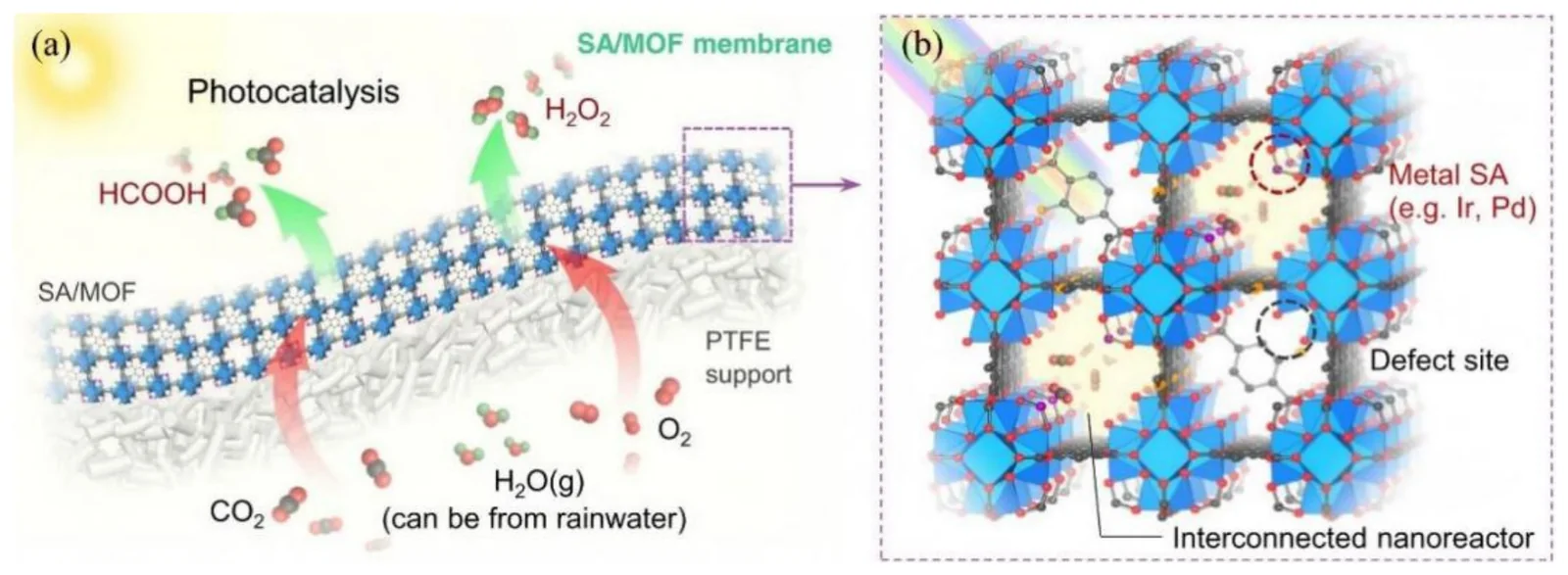
Schematic illustration and structural characterization of the SA/MOF membranes. (**a**) Humidified gases (e.g., CO_2_, O_2_) can be fed through the gas-permeable MOF/PTFE membranes and photocatalytically reduced to value-added chemicals (e.g., HCOOH and H_2_O_2_) under visible light irradiation and ambient conditions. (**b**) With controllable defect engineering, specific metal SAs (e.g., Ir and Pd) can be precisely anchored on the edges of the Zr_6_O_4_(OH)_4_(CO_2_)_12−x_ octahedral to act as programable catalytic centers for catalyzing different reactions, such as photocatalytic CO_2_-to-HCOOH and O_2_-to-H_2_O_2_ conversion. Adapted with permission from [34]. Copyright 2021 Springer Nature.

Hao et al. anchored single-atom Ir1 and Pd1 onto MOF (A-aUiO) nodes and assembled the resulting SA/MOF particles layer-by-layer onto a porous PTFE membrane (Figure 4). This gas–solid interface allows direct CO_2_ diffusion to the exposed single-atom sites, enabling CO_2_RR to HCOOH with near-100% selectivity and boosting the apparent quantum efficiency at 420 nm from 2.51% (Ir1/A-aUiO particles) to 15.76% (membrane)—a ~6-fold enhancement from improved gas accessibility. Using the same architecture, Pd1 sites catalyze the two-electron photoreduction of O_2_ to H_2_O_2_, achieving 10.4 mmol·gcat^−1^·h^−1^ in the membrane form, again surpassing the particulate catalyst. This design combines the highly dispersed, accessible active sites of homogeneous photocatalysts with the efficient product separation of heterogeneous systems, bridging the two paradigms [34].

A further advance in membrane-form photocatalytic CO_2_ reduction was demonstrated by Mu et al., who exploited the intrinsic electrical conductivity of the two-dimensional MOF Ni_3_HITP_2_ to construct 2D/2D composite thin films for CO_2_ photoreduction. Through vacuum filtration, negatively charged reduced graphene oxide (rGO) nanosheets and positively charged Ni_3_HITP_2_ particles were co-deposited via Coulomb interaction to form uniform Ni_3_HITP_2_/rGO heterostructure films. The intimate 2D/2D interface modulates the electronic structure of the Ni–N4 active centers via π–π stacking and electrostatic charge transfer, enhancing electron density at the catalytic Ni sites and accelerating CO_2_ activation. Under visible-light irradiation, the optimised composite film achieved a CO formation rate of 3.8 × 10^4^ μmol·h^−1^·g^−1^ film with a CO selectivity of 91.74%, representing a significant improvement over Ni_3_HITP_2_ alone (CO yield rate 3.45 × 10^4^ μmol·h^−1^·g^−1^, 97% selectivity). Crucially, the thin-film architecture enables extremely facile catalyst recovery: the film simply floats on the reaction solution and is collected by tweezers without any centrifugation or filtration, retaining high activity over multiple cycles. This work demonstrated for the first time that a conductive 2D MOF can be integrated with rGO into a recyclable thin-film cocatalyst format, opening a new pathway for membrane-based CO_2_ photoreduction using electrically conductive MOFs [32].

Photocatalytic CO_2_ reduction membranes have also been engineered around intrinsically catalytic, defect-rich MOF nanostructures rather than conductive 2D frameworks. Lin et al. immobilized defective Ni-MOF-74 (NMD) nanofragments, enriched in coordinatively unsaturated Ni sites, onto electrospun polyacrylonitrile (PAN) nanofibers to obtain a composite membrane with a uniform fish-scale microstructure (NMD/PAN NFMs); the resulting membrane achieved a CO production rate of 1641 μmol·g^−1^·h^−1^ with 91% selectivity, exceeding that of the powdered NMD catalyst, while the fibrous architecture allowed the membrane to be readily recovered and reused, retaining 71% of its initial efficiency after seven cycles—with the modest activity loss attributed to loss of NMD crystallinity rather than catalyst leaching [70].

A complementary strategy couples a conductive MOF film directly with a molecular catalyst and a photosensitizer. Yuan et al. fabricated a large-area, ultrathin conductive MOF (cMOF) film from copper and a catecholate ligand, then site-selectively immobilized a dinuclear-metal molecular catalyst (DMC) within the cMOF channels and perovskite quantum-dot photosensitizers on its outer surface to obtain a [DMC@cMOF]-PVK heterostructure film; using water as the electron donor, the resulting film photocatalyst achieved a CO yield of 133.36 μmol·g^−1^·h^−1^, far exceeding the photosensitizer alone or catalyst-photosensitizer combination alone, and a stacked ten-layer device further increased the areal CO yield eightfold over a powder-based reference device owing to the film’s high optical transparency [71].

Lanthanide-based MOFs (Ln-MOFs) differ from transition-metal MOFs through their 4f electronic configurations and higher, more variable coordination numbers, and have been explored in heterogeneous and photocatalytic applications, including photocatalytic H_2_ evolution [64,65]. Membrane-form implementations, however, remain far less mature than their powder counterparts and have mainly targeted luminescent sensing and pollutant removal rather than catalysis, as in the mixed-lanthanide polyMOF thermometric membrane [21] and Eu-MOF/PVDF mixed-matrix membranes for Cr_2_O_7_^2−^ enrichment and sensing [66]. Where photocatalysis has been demonstrated in membrane form, activity was modest: a Eu-MOF/polysulfone membrane degraded only about 21% of RhB and 14% of Congo red in 3 h under sunlight, indicating that active-site exposure and film design still need optimization [67]. Embedding pre-synthesized luminescent Ln-MOF crystallites in polymer matrices is currently the most accessible route to membrane-configured Ln-MOF materials, although compatibility between the MOF and the polymer is not guaranteed [68].

#### 3.2.2. Pollutant Degradation

Beyond CO_2_ conversion, photocatalytic MOF membranes have been applied to the degradation of persistent organic pollutants such as antibiotics and dyes, where the central engineering challenge is to combine efficient reactive-oxygen-species (ROS) generation with a membrane architecture that avoids catalyst loss into the treated water. We further supplement representative photo-Fenton/Fenton-like degradation cases.

Within Fenton and Fenton-like membranes, the role of the membrane can be graded by the extent of its participation in the reaction. In the simplest case, the membrane serves only as a carrier that immobilizes the catalyst: Cu-MOF-808 nanoparticles embedded in a PVDF matrix catalyzed Fenton degradation of tyrosol with minimal copper leaching, and weak MOF–polymer interactions were suggested to contribute to the improved stability [72]. At the next level, the membrane matrix also takes part in pollutant capture. A cellulose acetate/Fe-MOF-74 mixed-matrix membrane removed diclofenac (96.0%) through concerted adsorption and H_2_O_2_-activated Fenton oxidation, in which •OH, ^1^O_2_ and O_2_•^−^ were identified as reactive species [73]. At the highest level, the membrane integrates separation with catalysis. A g-C_3_N_4_ nanosheet membrane intercalated with MIL-100(Fe) coupled size-selective filtration with Fenton-like degradation, in which the intercalated MOF enlarged the interlayer spacing and accelerated Fe^2+^/Fe^3+^ electron transfer, giving ≥99% dye removal at a stable flux of 98.2 L·m^−2^·h^−1^·bar^−1^ together with self-cleaning ability [74].

Li et al. reported an in situ green synthesis of fibrous, leaf-like bimetallic ZIF nanocrystals directly grown on polyacrylonitrile (PAN) nanofiber membranes, yielding a ZIF/PAN composite that achieved high-performance degradation of the antibiotic norfloxacin; the hierarchical leaf-like morphology increased the exposed active-site density while the PAN nanofiber scaffold ensured the catalyst remained immobilized and recoverable across repeated filtration cycles [75]. Zhao et al. incorporated the iron-based photo-Fenton catalyst NH_2_-MIL-88B(Fe) into a polyvinylidene fluoride (PVDF) matrix via non-solvent-induced phase separation to form a mixed-matrix membrane, which coupled peroxymonosulfate (PMS) activation with membrane filtration to remove the antibiotic tetracycline hydrochloride from water; the amino-functionalized Fe-MOF filler simultaneously enhanced the membrane’s hydrophilicity and provided the catalytic sites required for PMS-driven oxidative degradation, illustrating how MOF selection can be used to tune both the separation and catalytic properties of the same membrane [76].

He et al. transformed waste PET plastic into a MOF precursor: Co-MOF nanorods synthesized by a one-pot solvothermal method were cross-linked and anchored to a PVDF substrate with polydopamine and assembled into a catalytic membrane by vacuum filtration [77]. Under visible light with peroxymonosulfate activation, the membrane degraded 97.63% of tetracycline hydrochloride within 25 min (k = 0.1645 min^−1^), retained more than 85% efficiency after five cycles and removed a range of other antibiotics, offering a low-cost route to MOF catalytic membranes from plastic waste.

Photothermal and photocatalytic mechanisms can also be combined within a single MOF membrane to accelerate self-cleaning during oil/water separation. Xiang et al. cross-linked UiO-66-NH_2_ particles with graphene oxide (GO) and polyvinyl alcohol (PVA) via suction filtration to obtain a PVA/GO@MOF membrane combining underwater superoleophobicity with light-driven self-cleaning; the membrane maintained a stable crossflow flux above 1250 L·m^−2^·h^−1^·bar^−1^ with over 99% emulsion-separation efficiency across 20 cycles, and its combined photothermal and photocatalytic effect degraded adsorbed organic foulants at 99.9% efficiency within 50 min, restoring flux without a separate chemical cleaning step; the membrane also retained its underwater oil contact angle above 150° after prolonged exposure to corrosive solutions, UV irradiation, ultrasound, abrasion, and repeated bending [36].

Chen et al. introduced NiFe-MOF/carbon-quantum-dot (CQD) nanocomposites into the polyamide layer by interfacial polymerization to construct a photocatalytic self-cleaning nanofiltration membrane, in which the CQDs improved filler hydrophilicity and dispersion while enhancing visible-light activity [78]. The optimal membrane reached a water permeance of 20.2 L·m^−2^·h^−1^·bar^−1^ (2.3 times that of a conventional thin-film composite membrane) with 98.2% Na_2_SO_4_ rejection, more than 86% rejection of various dyes and more than 93.4% tetracycline rejection over a wide pH range, and recovered more than 95% of its flux after five fouling–regeneration cycles under visible light.

A related nanocellulose-based strategy further illustrates how a biobased membrane scaffold can be combined with a Cu-MOF photocatalyst. Yin et al. deposited Cu-MOF micro/nanoparticles in situ onto the surface of a bacterial cellulose (CB) membrane, then modified the surface with stearic acid (STA) to obtain a CB/Cu-MOF/STA composite membrane that unites superhydrophobic oil/water separation, photocatalytic dye degradation, and anti-biofouling in a single material; the multifunctional membrane simultaneously separated oil/water mixtures with high efficiency, photocatalytically degraded model dyes under irradiation, and resisted bacterial biofouling, illustrating a route toward multifunctional, biomass-derived catalytic MOF membranes for sewage remediation [79].

#### 3.2.3. Reduction and Removal of Heavy Metals

The primary valence states of chromium are Cr(III) and Cr(VI). Compared to the less toxic Cr(III), Cr(VI) is a hundred times more toxic. Its high toxicity can cause harm to the human body’s respiratory and immune systems, and may also lead to genetic mutations, as well as kidney and liver damage. Consequently, the direct discharge of chromium-containing wastewater without treatment poses a serious threat to human health and the ecological environment. As a highly efficient, energy-saving and environmentally friendly emerging technology, photocatalysis holds great promise; consequently, the design of highly efficient catalytic materials for the photocatalytic reduction of Cr(VI) is of significant importance.

To address the difficulty in separating traditional powdered photocatalysts from water, Du et al. employed the reaction seeding method to prepare UiO-66-NH_2_ (Zr/Hf) MOF membrane photocatalysts on an α-Al_2_O_3_ substrate. This film achieved highly efficient reduction of Cr(VI) under both simulated and natural sunlight, whilst exhibiting excellent chemical and aqueous stability; even after 20 cycles, the Cr(VI) reduction efficiency remained above 94%, providing a new pathway for the highly efficient photocatalytic reduction of heavy metals [31].

#### 3.2.4. Efficient Hydrogen Production

With the depletion of fossil fuels and the growing urgency of environmental protection, the demand for new, clean solutions is increasing. As a key technology for directly converting solar energy into hydrogen, photocatalytic water splitting offers a potential solution to the global energy crisis. Metal–organic framework (MOF)-based photocatalysts offer highly customizable structures due to the diversity of their organic ligands and metal nodes, enabling the light absorption spectrum to be extended from ultraviolet to visible and even infrared light. Furthermore, the framework’s compatibility facilitates the integration of photosensitizers and active sites. Consequently, selecting MOFs as photocatalysts offers significant advantages [37].

In typical photocatalytic hydrogen production experiments, MOFs are often dispersed in water in powder form to form a suspension system. However, powdered catalysts present challenges such as difficulty in recovery and high additional costs. To optimize the hydrogen production performance of MOF-based photocatalysts, Zhao et al. [30] prepared a copper(II)-based metal–organic framework film (MOF-199/Ni) with high photocatalytic hydrogen production performance via an electro-deposition method. The 50 nm MOF-199 nanoparticles were uniformly dispersed and tightly anchored onto a nickel foam substrate, providing abundant copper active sites with unsaturated coordination for the photocatalytic reaction. This resulted in high hydrogen production rates, and the catalyst was reusable with no significant loss of hydrogen production activity. This strategy of ‘coating MOFs onto porous substrates’ is also universally applicable; it can be used to prepare novel, highly active, and easily recoverable photocatalysts for hydrogen production, and offers new opportunities for the development of heterogeneous catalytic systems across multiple fields.

The ordered framework structure of MOFs also provides an ideal platform for defect engineering; however, defect engineering typically compromises the stability of MOF crystals, resulting in insufficient stability for hydrogen production. Therefore, the design of MOF membranes holds promise for the development of photocatalysts with excellent stability and a long hydrogen production lifespan. Liu et al. [37] were the first to evaluate the long-term photocatalytic stability of lattice-defect-containing MOFs by in situ growing a zirconium-based, water-stable MOF membrane (Pt@UNPM/Pt) rich in lattice defects, with large pore sizes and loaded with trace amounts of Pt on a polymer substrate. The lattice-defect-rich structure, combined with Pt modification, synergistically enhances photocatalytic activity by shortening the electron transfer distance, improving electron transfer efficiency, and effectively suppressing electron–hole recombination; furthermore, it exhibits excellent stability and a long hydrogen production lifespan.

Building on this same design philosophy, Liu et al. [80] subsequently constructed a three-dimensional Pt@UCM/CdS network by first growing a Pt-doped UiO-66-NH_2_ MOF membrane (UCM) in situ on a carboxyl-modified polypropylene fibrous substrate, then depositing CdS nanoparticles onto the MOF surface via a liquid-phase sulfidation step. The resulting interpenetrating MOF–CdS network forms an S-scheme heterojunction that provides multiple charge-transport pathways while the three-dimensional architecture mechanically buffers CdS against photocorrosion and prevents collapse of the MOF scaffold. This membrane achieved a hydrogen evolution rate of 47.45 mmol·m^−2^·h^−1^, far exceeding that of the Pt@UCM membrane alone; it sustained catalytic operation for 1100 h in pure water with 75% of initial activity recoverable after ultrasonic cleaning, and remained stable for 600 h in natural seawater—underscoring that membrane architecture can extend photocatalyst lifetime by orders of magnitude relative to suspended powders even in complex, real-world aqueous matrices.

Protecting an intrinsically unstable MOF photocatalyst within a polymer matrix offers a complementary strategy for durable hydrogen production. Gontarek-Castro et al. embedded copper-modified NH_2_-MIL-125(Ti) particles within a polyvinylidene fluoride (PVDF) matrix to form a photocatalytic composite membrane, systematically varying the MOF loading from 1 to 10 wt%; the 10 wt% membrane achieved the highest H_2_ production rates of 389.1 μmol·h^−1^·m^−2^ in an aqueous electrolyte and 466.0 μmol·h^−1^·m^−2^ in acetonitrile, with the PVDF matrix substantially improving the structural stability of the otherwise moisture-sensitive MOF, which retained its crystalline integrity after 20 h of continuous photoreaction, underscoring that polymer encapsulation can rescue intrinsically unstable MOF photocatalysts for practical membrane-based hydrogen generation [81].

The sixteen photocatalytic membranes in Table 2 span more than two orders of magnitude in reported CO_2_-reduction yield (133 μmol·g^−1^·h^−1^ for [DMC@cMOF]-PVK versus 3.8 × 10^4^ μmol·h^−1^·g^−1^ for Ni_3_HITP_2_/rGO), but these figures are not directly comparable: they are normalized to different bases (per gram of catalyst, per unit membrane area) and obtained under different light sources and intensities, so the apparent two-order-of-magnitude spread likely reflects methodological heterogeneity as much as genuine catalytic superiority. Apparent quantum efficiency—the one metric that would allow a photon-normalized comparison—is reported for only a single system (Hao et al.’s SA/MOF, 15.76% at 420 nm), leaving the rest of the CO_2_-reduction subset effectively unrankable on a like-for-like basis. A further gap concerns the field’s central claim that membrane architecture enables catalyst recovery and reuse: this is explicitly measured with multi-cycle data for only about half of the entries (e.g., Ni_3_HITP_2_/rGO, NMD/PAN NFMs), while others (SA/MOF, [DMC@cMOF]-PVK) assert the recyclability advantage without reporting cycling data at all. The hydrogen-production subset is the clear exception and arguably the strongest evidence in the entire review: Liu et al.’s Pt@UCM/CdS network sustained 1100 h of operation in pure water and 600 h in natural seawater, and Gontarek-Castro et al. demonstrated that PVDF encapsulation can rescue an intrinsically moisture-unstable MOF over 20 h of continuous photoreaction. This degree of long-duration, real-matrix testing has no counterpart in the CO_2_-reduction or pollutant-degradation subsections, where cycling data rarely exceed five to seven cycles or tens of minutes to hours. The root cause is again a lack of standardized reporting—light source, reactor geometry, and catalyst loading vary freely across studies—but within that constraint, hydrogen production stands out as the reaction class where membrane-specific durability claims are best substantiated, and CO_2_ reduction as the one where they are most often assumed rather than demonstrated.

Time-resolved photoluminescence (TRPL) and transient absorption spectroscopy are widely used to quantify excited-state lifetimes and charge-carrier dynamics in MOF photocatalysts, and they offer direct insight into charge-transfer processes and trap-state distributions [82]. Surface-bound intermediates can be followed by in situ DRIFTS, while radical species and paramagnetic centres are identified by EPR [82]; in situ FTIR and Raman spectroscopy complement these for surface species [83]. Nevertheless, most in situ techniques remain relatively simple, with limited sensitivity and spatiotemporal resolution, whereas charge transfer, reaction intermediates and surface species evolve dynamically across multiple time and length scales [83]. For membrane photocatalysts, a further caveat is that many of these measurements are made on powders or dry films rather than on membranes in an operating reactor.

### 3.3. Thermocatalytic Applications

Thermocatalysis is an important branch of catalysis, and thermal energy materials with catalytic properties are referred to as thermocatalysts. Compared with conventional thermocatalytic oxides, MOFs offer greater structural diversity and tunability, and can be coated onto substrates such as carbon fiber fabric to form functional thermocatalytic filters. In membrane form, they can further couple catalytic activity with selective permeation [17,18]. Currently, MOFs and MOF-derived materials have been widely employed in thermal CO_2_ hydrogenation, where the framework serves either as a support or as a sacrificial precursor [84,85,86]. The following section details the thermocatalytic applications of MOF membranes across different temperature ranges through several examples.

#### 3.3.1. Low-Temperature Thermocatalytic Antibacterial Applications

High-efficiency antimicrobial activity at low-temperature conditions is a core requirement in fields such as medical protection and outdoor water treatment. Traditional antimicrobial technologies suffer from inherent limitations, including high energy consumption, poor stability, and a tendency to cause secondary contamination. These drawbacks make it difficult to meet the demands for long-lasting performance and low energy consumption in such scenarios. In contrast, the low-temperature thermocatalytic antimicrobial system based on MOF membranes achieves long-lasting antimicrobial activity through the generation of active substances under mild conditions.

**Figure 5:**
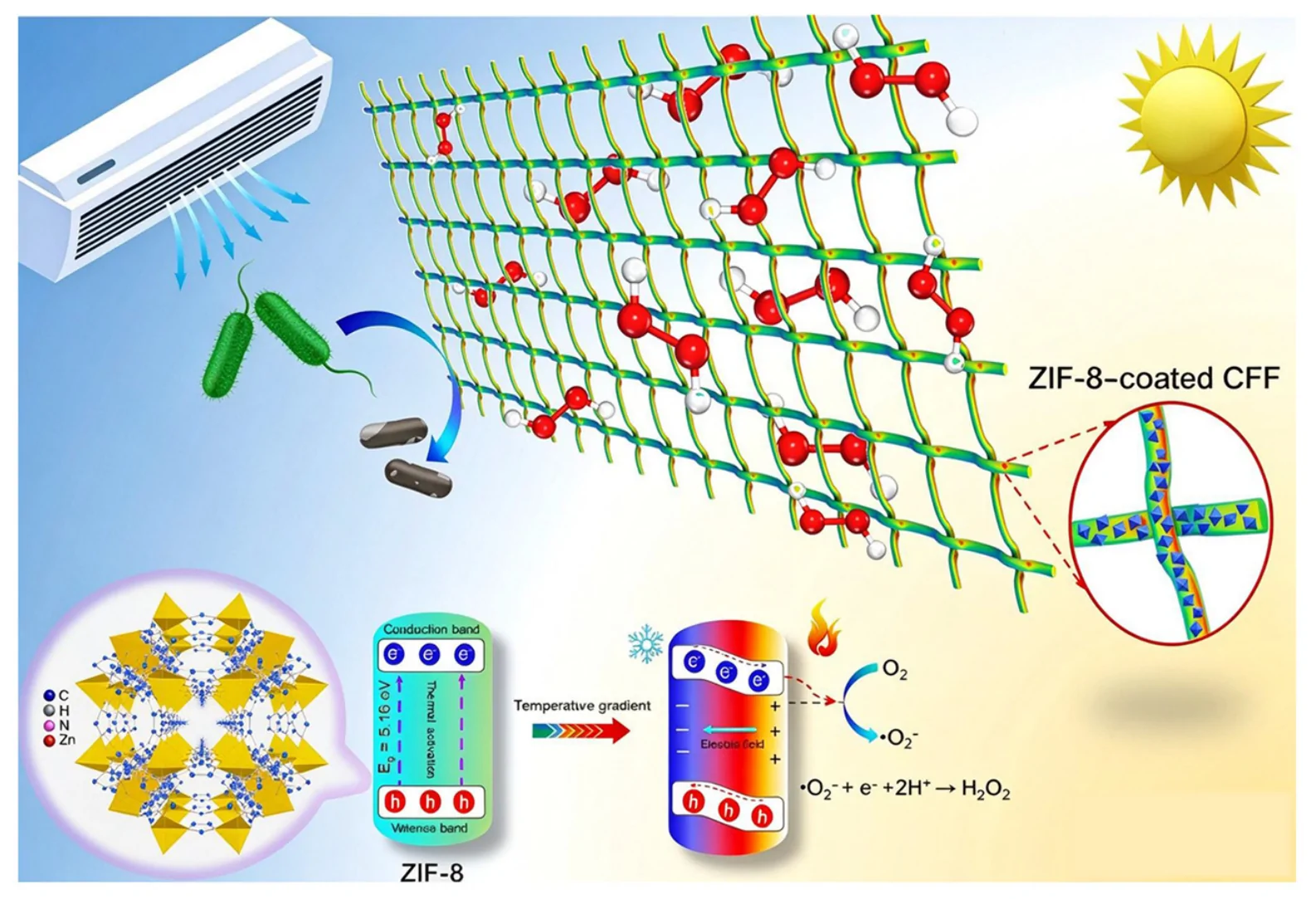
Schematic illustration of the ZIF-8–coated carbon fiber fabric (CFF) system for H_2_O_2_ generation driven by temperature gradient. The inset shows the ZIF-8 coating on CFF and the proposed mechanism of O_2_ reduction to H_2_O_2_. Adapted with permission from [40]. Copyright 2025, AAAS.

Pal et al. proposed a method for fabricating an antibacterial membrane by selecting Zn-based ZIF-8 to prepare a ZIF-8@CFF composite filter. ZIF-8 nanoparticles (NPs) were coated onto carbon fiber fabric (ZIF-8@CFF) for practical applications (Figure 5), and the ZIF-8@CFF was employed as a thermocatalytic filter utilizing indoor temperature differentials to mimic real-world conditions [40]. When a temperature difference of 5–10 °C occurs in the environment (such as the diurnal temperature variation), thermal energy drives the Zn^2+^ active sites in ZIF-8 to catalyze a redox reaction between O_2_ in the air and H_2_O molecules in the environment, generating hydrogen peroxide (H_2_O_2_)—a highly effective antimicrobial agent. Importantly, the generated H_2_O_2_ acts directly on pathogenic bacteria upon contact without requiring additional diffusion, thereby achieving high antimicrobial activity.

Subsequent studies demonstrated that the ZIF-8@CFF composite filter membrane achieved an inactivation rate of up to 96% against two common pathogenic bacteria, Escherichia coli and Staphylococcus aureus. More notably, after 30 days of operation, the ZIF-8 crystals remained uniformly dispersed on the CFF surface without obvious detachment or dissolution, indicating remarkable stability [40]. This performance not only addresses the shortcomings of traditional antimicrobial technologies but also offers unlimited possibilities for achieving long-lasting antimicrobial protection in low-temperature environments and developing energy-efficient disinfection systems.

Beyond thermocatalytic H_2_O_2_ generation, MOF membranes have also been explored as antibacterial platforms through intrinsic metal-ion release. Zhou et al. grew Cu-BTC (HKUST-1) directly on poly(ionic liquid)-based electrospun fibrous membranes to fabricate a multifunctional wound dressing combining air permeability, moisture absorption, and antibacterial activity; the in situ grown Cu-BTC layer provided a sustained source of bactericidal Cu^2+^ ions and reactive oxygen species at the fiber surface, complementing the temperature-gradient-driven H_2_O_2_ generation strategy described above and underscoring the versatility of MOF membranes across different antibacterial mechanisms [38].

#### 3.3.2. Medium-Temperature Catalytic Oxidative Desulfurization

Removing organosulfur compounds from liquid fuels via catalytic oxidation is another medium-temperature thermocatalytic reaction for which MOF membranes are well suited, since the membrane form directly addresses the recurring challenge of separating a powdered catalyst from a viscous fuel matrix after reaction.

Faria et al. directly compared three UiO-66/PVDF mixed-matrix membranes, prepared by different routes from the same MOF and polymer chemistry, for the catalytic oxidative desulfurization of a model fuel, providing an unusually clean illustration of how fabrication route governs active-site accessibility. In the conventional blending route, UiO-66 powder was first synthesized solvothermally and then dispersed into a PVDF casting solution; the resulting membrane completely encapsulated the MOF crystallites beneath a continuous PVDF layer with no crystallites exposed at the surface, and achieved only 54% desulfurization in 2 h because most active sites remained inaccessible to the fuel. By contrast, a one-step route that co-dissolved the zirconium salt, linker, and PVDF and allowed the MOF to crystallize in situ as the solvent evaporated at 90 °C produced a defect-rich membrane (Cl/Zr ratio of 0.10) with ~100 nm UiO-66 crystallites distributed throughout and exposed across the membrane thickness; this membrane reached 96% desulfurization in 2 h, matching the performance of pristine UiO-66 powder, and retained its activity with no detectable metal leaching over five reuse cycles. Introducing a formic acid modulator into the same one-step process yielded larger, more crystalline particles (~470 nm) with a lower defect density (Cl/Zr ratio of 0.04); this modulated membrane was structurally more robust but reached only 69% desulfurization, since the reduction in framework defects also removed the coordinatively unsaturated sites responsible for catalytic oxidation. Together, these three membranes—prepared from identical starting materials but different assembly sequences—demonstrate that catalyst encapsulation, defect density, and crystallite exposure, rather than MOF loading or chemistry alone, are the primary levers governing the catalytic performance of mixed-matrix MOF membranes [39].

#### 3.3.3. Medium-Temperature Selective Catalytic Semihydrogenation of Alkynes

Currently, the selective hydrogenation of alkynes to alkenes is a crucial step in the production of polymers and fine chemicals. The key challenge lies in avoiding over-hydrogenation to alkanes while simultaneously ensuring high catalytic activity and selectivity [87]. Among the numerous semi-hydrogenation catalysts developed to date, noble metal-based catalysts exhibit higher catalytic activity compared to non-metal-based catalysts; however, they often suffer from low selectivity due to their propensity for over-hydrogenation [88,89].

To date, however, MOF-based semihydrogenation catalysts have been evaluated almost exclusively as dispersed powders [88,89], and, to our knowledge, no intact MOF membrane has yet been applied to this reaction. The membrane format nevertheless appears well matched to its central selectivity problem: a continuous MOF layer could confine metal nanoparticles against sintering, while molecular sieving and transmembrane control of the H_2_ supply could shorten the residence time of the alkene product near the active sites and thereby suppress over-hydrogenation. Demonstrating such a membrane—with matched powder controls, time-on-stream data, and post-reaction structural characterization—therefore represents a clear opportunity for future work.

#### 3.3.4. High-Temperature Thermocatalytic Conversion: Current Status and Outlook

While low- and medium-temperature thermocatalytic MOF membranes have reached considerable maturity, the application of MOF membranes under high-temperature conditions (above 200 °C) remains a largely unexplored frontier, and represents a critical gap in the current literature. At elevated temperatures, most conventional MOFs undergo structural decomposition due to ligand combustion and loss of coordinated solvent; however, thermally robust MOF families—including zirconium-based UiO-66/UiO-67, MIL-53, and certain zeolitic imidazolate frameworks (ZIFs)—retain crystallinity and porosity up to 300–400 °C [90,91,92], placing them within the operating window of industrially important reactions such as CO_2_ methanation (200–300 °C), methanol synthesis (200–250 °C) [93], selective VOC oxidation (200–350 °C), and alkane dehydrogenation [94]. In these contexts, an MOF membrane could in principle serve a dual function: acting as a catalytic reactor while simultaneously removing thermodynamic by-products (e.g., water in methanol synthesis) through size-selective permeation, thereby shifting equilibrium conversion beyond the thermodynamic limit of conventional fixed-bed reactors. MOF-derived catalysts—in which the organic ligand is pyrolyzed to yield metal oxides or metal-carbon composites while partially retaining the parent MOF’s morphology and pore architecture—have already demonstrated competitive activity in CO_2_ hydrogenation and selective oxidation reactions [95], suggesting a viable pyrolytic route to thermally stable MOF-derived membrane reactors. The translation of intact MOF membranes into high-temperature membrane reactor configurations therefore represents a particularly promising and underexplored direction, one that may ultimately bridge the gap between the structural designability of MOFs and the demanding thermal conditions of large-scale industrial catalysis.

The five thermocatalytic membranes in Table 3 are fewer in number but, in one respect, more methodologically rigorous than either the electro- or photocatalytic sets: Faria et al.’s three UiO-66/PVDF desulfurization membranes were prepared from identical MOF and polymer chemistries, differing only in assembly sequence, providing a rare genuine internal control. The resulting monotonic relationship—54% desulfurization when the MOF is fully encapsulated, rising to 96% when defect-rich crystallites are exposed at the surface (Cl/Zr = 0.10), then falling to 69% when a crystallization modulator reduces defect density (Cl/Zr = 0.04)—is the clearest fabrication-to-performance causal chain reported anywhere in this review, and should be regarded as a methodological template other subfields would benefit from adopting. The antibacterial entries (ZIF-8@CFF, Cu-BTC/PIL), by contrast, each stand as isolated data points without a matched-condition comparator, so while their absolute performance is strong (96% bacterial inactivation), no equivalent structure–activity conclusion can be drawn from them. It is also notable that alkyne semihydrogenation, which runs in a medium-temperature window comparable to that of oxidative desulfurization (~90 °C), has not yet been demonstrated with an intact MOF membrane (Section 3.3.3)—an unexplored opportunity. Most fundamentally, high-temperature thermocatalysis (>200 °C) has not yet been examined with experimental membrane data, so Section 3.3.4 is necessarily prospective (Table 4); closing this gap is arguably the most pressing task for thermocatalytic MOF membranes. Finally, even within the populated low- and medium-temperature regimes, reported testing durations are short (often single 2 h runs) relative to the 1100 h benchmark achieved in photocatalytic hydrogen production, indicating that long-duration operational stability—not just peak conversion—remains the key unaddressed question for thermocatalytic MOF membranes.

TGA-DSC provides rapid preliminary screening of thermal decomposition thresholds for MOF-membrane specimens under inert or oxidative atmospheres, but these measurements are performed on static samples and cannot replicate flowing mixed reactant gases. In situ variable-temperature PXRD tracks crystallinity changes under gas flow, although commercial sample cells limit high-pressure experiments such as hydrogenation. In situ XAS is better suited to the question that matters most for thermocatalytic MOF membranes, namely whether the catalytic species are intact framework nodes or thermally generated metal or metal-oxide nanoparticles. In situ XAS, often combined with PXRD, has been used to follow changes in the coordination environment of MOF nodes during thermal activation and to monitor the formation of metal nanoparticles within MOFs, for example, Pd in UiO-67, as well as carbide species formed under reaction conditions [96]. This technique nevertheless requires synchrotron access and elaborate data-fitting workflows. Post-reaction XPS, SEM-EDX and ICP-MS are routinely used to detect metal leaching and morphological changes in spent catalysts, but they are post-mortem tools and cannot capture dynamic structural changes during the reaction itself.

Long-term time-on-stream stability data are scarce for thermocatalytic MOF membranes. Most studies report only short-batch conversion and overlook gradual ligand loss, sintering and metal leaching under thermal stress. Experimental examples of intact MOF membranes operating above 200 °C are nearly absent; most high-temperature tests employ MOF-derived materials, motivating the development of thermally robust intact-MOF membrane reactors. Few works quantify surface-area loss upon MOF incorporation into composite membranes, so further quantitative studies are needed to map trade-offs between mechanical robustness, accessible active site and mass transport. Multi-tasking thermocatalytic MOF-membrane platforms are rarely explored. Furthermore, lab evaluations rarely employ industrially relevant feed gas containing steam and sulfur poisons, limiting real-world translatability of laboratory results.

### 3.4. General Discussion: Three Functional-Integration Modes, Trade-Offs and Material Distinction for Catalytic MOF Membranes

Across electrocatalysis, photocatalysis, and thermocatalysis surveyed within this review, a unified design logic emerges that holds independently of the external energy input source (electricity, light, or thermal energy). Catalytic functionality within MOF-based membranes arises via one of three distinct functional-integration modes, which serve as the organizing framework linking fabrication choices to final catalytic performance.

The first mode relies on intrinsic framework active sites, where catalysis originates directly from metal nodes, coordinatively unsaturated centers, or defect sites belonging to the intact crystalline MOF lattice. Representative examples include Ni-, Cu-, and Zr-based frameworks applied in electrocatalytic hydrogen evolution, photocatalytic CO_2_ reduction, and oxidative desulfurization. Under this scenario, membrane fabrication priorities centre on preserving the crystallographic integrity of the parent MOF phase.

The second mode corresponds to guest species incorporation. Metal nanoparticles, single-atom sites, molecular catalysts, or secondary semiconductor phases are embedded and confined within MOF pore spaces. Here the MOF lattice predominantly acts as a confinement matrix that modulates guest dispersion, suppresses particle sintering, and tunes mass-transport behavior, rather than supplying the primary catalytic sites. Single-atom-loaded MOF membranes fall into this category [34,72]. Note that guest-loaded systems activated by pyrolysis may undergo partial framework decomposition and thus need to be classified as MOF-derived materials post-treatment. Fabrication in this case requires precise control over guest location and size distribution while preventing aggregation during membrane processing.

The third mode is non-catalytic microenvironment coating. In this configuration, the MOF layer itself possesses negligible intrinsic catalytic activity. It is conformally deposited onto a pre-functionalised catalyst or electrode exclusively to tailor local interfacial conditions: regulating reactant accumulation, proton-ion transport, and intermediate stabilization at solid–liquid or solid–gas boundaries. Well-documented examples are UiO-66-coated Ag electrodes for electrochemical CO_2_ reduction and MIL-53(Al)-modified LiNbO_3_ for electrochemical nitrogen reduction. Defect-free, uniform thin-film coverage over the underlying substrate represents the key fabrication target for this class of membranes.

These three integration modes are not mutually exclusive; hybrid systems combining two modes can also be encountered in published literature. Regardless of the integration pathway, membrane geometry delivers unique benefits unavailable for suspended powder MOF catalysts, including interfacial reactant enrichment, tunable transmembrane mass transport, integrated reaction-separation capability, and facile macroscopic catalyst recovery without centrifugation or filtration. Nevertheless, constructing catalytic MOF membranes also introduces inherent performance penalties. Polymer matrices or substrate interfaces can partially block exposed active sites and reduce accessible specific surface area relative to fine MOF powders, while interfacial pinhole defects may produce undesired reactant bypass. Whether membrane-derived advantages outweigh these drawbacks depends heavily on fabrication quality, membrane thickness, and the targeted catalytic application.

It is also necessary to keep in mind the material distinction between intact MOF membranes and MOF-membrane-derived catalysts raised at the opening of Section 3. Direct quantitative performance comparisons should be made cautiously between systems retaining the original MOF crystal framework and sacrificial-template-derived materials that lose their organic linkers upon thermal post-treatment, given their fundamentally different active-site origins and stability evaluation criteria.

## 4. Summary and Outlook

This review has surveyed catalytic MOF membranes in electrocatalysis, photocatalysis, and thermocatalysis, organized by how catalytic function is integrated with the membrane: intrinsic framework active sites, guest species incorporation, and non-catalytic microenvironment coating. In electrocatalysis, non-catalytic MOF layers on Ag and LiNbO_3_ improved CO_2_ and N_2_ reduction by regulating reactant delivery at the electrode interface [22,26], whereas the best-performing water-splitting electrodes, Co_2_P/CoP@C and the protic-salt-cured NiO-based membrane, were MOF-derived or only partially crystalline [55,56]. In photocatalysis, MOF membranes supported CO_2_ reduction with near-unity HCOOH selectivity [34], pollutant degradation, Cr(VI) reduction, and H_2_ evolution, with the most convincing durability evidence for Pt@UCM/CdS, which operated for 1100 h in pure water and 600 h in natural seawater [80]. In thermocatalysis, three UiO-66/PVDF membranes showed that catalyst encapsulation and defect density govern desulfurization activity [39], whereas alkyne semihydrogenation and high-temperature reactions have not yet been demonstrated with intact MOF membranes. Across the three regimes, membrane geometry offers reactant enrichment, product separation, and macroscopic catalyst recovery that suspended powders cannot provide [17,18].

At the same time, the evidence base remains uneven. Most results are laboratory-scale, cycling and time-on-stream data are often short, and the two best-benchmarked electrocatalytic systems depart from the definition of an intact MOF membrane. High-temperature operation (>200 °C) of intact MOF membranes has no experimental membrane data to date. Distinguishing intact membranes from MOF-derived catalysts is therefore a prerequisite for meaningful comparison, and future reports should state which class a material belongs to and support the assignment with post-reaction XRD, spectroscopy, and leaching analysis.

Several directions follow from the preceding sections. First, structural stability under aqueous, acidic, oxidative, and high-current conditions remains the central materials challenge. Defect-tolerant frameworks, polymer or inorganic protection, and membrane architectures that shield the framework are promising routes, as the PVDF-protected NH_2_-MIL-125(Ti) membrane illustrates [81]. Operando Raman, X-ray absorption, and synchrotron-based diffraction should be adopted routinely to follow structural evolution under working conditions, rather than relying solely on post-reaction ex situ analysis [61,96]. Second, catalytic conversion and membrane permeation are still usually evaluated separately; future work should co-optimize them in configurations that exploit integrated reaction–separation, such as thermocatalytic membrane reactors for equilibrium-limited reactions [18]. Third, most reported fabrication relies on batch solvothermal synthesis, which limits scale-up. Continuous-flow and milder synthetic routes, standardized substrate pre-treatment, and large-area defect-free coverage need systematic attention [17]. Finally, computational screening and data-driven methods may help navigate the large chemical space of MOFs, provided that standardized experimental datasets become available.

Consistent reporting is the community-wide prerequisite for all of these steps. As highlighted in Section 3, catalytic metrics are normalized to different bases (per catalyst mass or per membrane area), measured under heterogeneous irradiation or electrochemical conditions, and obtained in different reactor geometries, which prevents reliable cross-paper comparison. Table 5 lists the minimum experimental variables we recommend future studies report, covering material characterization, testing parameters, performance metrics, and stability.

## Figures and Tables

**Table 1:** (**A**). Fabrication and structural features of electrocatalytic MOF membranes. (**B**). Catalytic performance and testing conditions of electrocatalytic MOF membranes.

(**A**)					
Membrane/Catalyst	Fabrication route	MOF type	Key structural features	Catalytic application	Ref.
Co-MOF@CoP/carbon fiber	In situ growth + phosphorization	Co-MOF	Continuous nanofilm; intimate MOF-derived CoP-carbon contact; porous membrane	Overall water splitting (OER/HER)	[54]
Co_2_P/CoP@C/graphite	Cathodic electrodeposition + full phosphorization (MOF-templated)	O-coordinated Co-MOF (fully decomposed)	MOF-templated heterostructure; conductive pathway; Co_2_P/CoP interface	HER & OER	[55]
NiO/dmea MOF membrane	Protic-ionic-liquid (dmea/dema) + thermal curing	NiO-based, Pbca, diamondoid topology	Smooth homogeneous crystallites; strong Ni–N coordination	HER	[56]
Scalable MOF films (bionic mineralization)	Metal corrosion + biomimetic mineralization on 7 metals; inkjet patterning	16 MOFs (carboxylate, N-heterocycle, phenolate, phosphonate)	μm-to-m patternable films; mild synthesis	HMF oxidation coupled with HER	[24]
UiO-66/Ag	Solvothermal growth on Ag	UiO-66 (non-catalytic coating)	Insulating MOF membrane; TMA post-modification for electrostatic steering	CO_2_RR to CO	[22]
Fe-MOF-525/ITO	Meniscus-guided solution shearing + Fe^3+^ metalation	MOF-525 (porphyrin)	~20% porphyrin metalated; intact framework	CO_2_RR to CO	[23]
CALF-20/PSF on gas diffusion electrode	MOF filler in PSF coated on GDE	CALF-20 (non-catalytic coating)	CO_2_-philic mixed-matrix layer; CO_2_/N_2_ selectivity ~40	CO_2_RR to CO from dilute CO_2_	[27]
MOF molecular-sieve membrane/Bi electrode	Self-supporting MOF membrane integrated with diffusion and Bi catalyst layers	MOF-based (non-catalytic sieving layer)	CO_2_ enrichment; SO_2_/NO/O_2_ rejection	CO_2_RR to HCOOH from air/flue gas	[28]
Cu(I)–Cu-HHTP film/Cu(100)	In situ electrochemical assembly + pre-reduction	Cu-HHTP (2D conductive)	Cu_2_O clusters confined in framework cavities; Cu(I)/Cu^0^ dual sites	CO_2_RR to C_2_ products	[25]
MIL-53(Al)/LiNbO_3_	Ultrathin in situ growth	MIL-53(Al) (non-catalytic coating)	Al-O-Al N_2_-binding sites; hydrophobic barrier; thickness-tunable	NRR to NH_3_	[26]
MOF-808 bipolar membrane	Interlayer assembly in bipolar membrane	MOF-808	Catalytic interlayer between cation/anion exchange layers	Water dissociation + DMNP hydrolysis	[57]
Cu-TCPP/MOF/MXene/AgNWs	Blend into polyethersulfone	Cu-TCPP	Conductive filler network; ultrafiltration membrane	Dye degradation	[58]
CuPbZn-BTC membrane	Electrochemical decomplexing recovery	Mixed-metal CuPbZn-BTC	Abundant oxygen vacancies + Lewis acid sites	Antibiotic degradation	[59]
Ni/Co-BTC@carbon felt cathode	Cathodic electrodeposition	Ni/Co-BTC	Bimetallic MOF on porous carbon felt; enhanced ORR/H_2_O_2_ generation	Bio-electro-Fenton degradation	[29]
MIL-88B(Fe)–LIG cathode	MIL-88B(Fe) integrated on laser-induced graphene	MIL-88B(Fe)	Fe-MOF on porous LIG; in situ H_2_O_2_ generation	MFC-Fenton antibiotic degradation	[60]
(**B**)					
Membrane	Reaction	Testing conditions	Key performance metrics	Stability	Ref.
Co_2_P/CoP@C	HER/OER	Alkaline water splitting	Overpotential 146 mV (HER), 365 mV (OER) at 100 mA·cm^−2^	Not reported	[55]
NiO/dmea	HER	Neutral electrolyte	Tafel 30 mV·dec^−1^; 590 mA·cm^−2^; TOF 1.8408 × 10^18^ s^−1^	168 h at 1 A·cm^−2^	[56]
Bionic MOF films	HMF oxidation + HER	64 cm^2^ electrolyzer	3.0 A at 1.63 V	Not reported	[24]
UiO-66/Ag	CO_2_RR	CO_2_-saturated electrolyte	Enhanced CO selectivity & activity vs. bare Ag	Not reported	[22]
Fe-MOF-525	CO_2_RR	CO_2_-saturated organic electrolyte	Superior CO production vs. bare ITO/non-metalated film	Not reported	[23]
CALF-20/PSF GDE	CO_2_RR to CO	10 vol% CO_2_ feed, MEA	j(CO) ≈ 42.7 mA·cm^−2^ (3× bare GDE); FE(CO) ≈ 70%	>20 h	[27]
MOF sieve membrane	CO_2_RR to HCOOH	Flue gas or air feed, acidic	Up to 9000 mA, FE(HCOOH) ≈ 100% (flue gas)	Not reported	[28]
Cu-HHTP film	CO_2_RR to C_2_	Electroreduction on Cu(100) support	C_2_ selectivity 72.6%; >300 mA·cm^−2^	>50 h	[25]
MIL-53(Al)/LiNbO_3_	NRR	Aqueous electrolyte	NH_3_ yield 45.25 μg·h^−1^·mg^−1^_cat.; improved FE	Not reported	[26]
MOF-808 bipolar	Water dissociation	Bipolar membrane under bias	93.3% dissociation in 1 h; DMNP 95% in 128 min; >90% after 20 cycles	20 cycles	[57]
Cu-TCPP/MXene/AgNWs	Dye degradation	5 V bias, ultrafiltration	>90% degradation of 4 dyes in 5 h; >98% Congo red rejection	Flux ~3× vs. polymer	[58]
CuPbZn-BTC	Antibiotic degradation	Electrochemical	92–99% removal, no added oxidant	Not reported	[59]
Ni/Co-BTC@CF	Bio-electro-Fenton	Bisphenol S 1–10 mg·L^−1^, 24 h	98/84/41% removal; 133.6 mW·m^−2^	Not reported	[29]
MIL-88B(Fe)–LIG	MFC-Fenton degradation	Ciprofloxacin, 180 min	95.3% removal; 42.4% TOC; 90.7% in real wastewater	Minimal Fe leaching	[60]

**Table 2:** (**A**) Fabrication and structural features of photocatalytic MOF membranes. (**B**) Catalytic performance and testing conditions of photocatalytic MOF membranes.

(**A**)					
Membrane/Catalyst	Fabrication route	MOF type	Key structural features	Catalytic application	Ref.
SZIF-67/PAN NFMs	In situ growth on electrospun PAN + thermal stabilization	ZIF-67	Visible-light responsive; photothermal conversion	CO_2_ reduction	[35,42]
SA/MOF/PTFE (Ir_1_, Pd_1_)	Layer-by-layer deposition	A-aUiO (defect-engineered)	Single atoms anchored on Zr_6_O_4_(OH)_4_(–CO_2_)_12−x_ edges; gas-permeable	CO_2_→HCOOH; O_2_→H_2_O_2_	[34]
Ni_3_HITP_2_/rGO film	Vacuum filtration (Coulomb co-deposition)	Ni_3_HITP_2_ (2D conductive)	Intimate 2D/2D heterointerface; π–π stacking; Ni–N_4_ active centers	CO_2_ reduction	[32]
NMD/PAN NFMs	Electrospinning	Defective Ni-MOF-74	Unsaturated Ni sites; fish-scale microstructure	CO_2_ reduction	[70]
[DMC@cMOF]-PVK film	cMOF film + molecular catalyst + QD photosensitizer	Conductive Cu-catecholate cMOF	DMC in channels; perovskite QDs on surface; transparent film	CO_2_ reduction	[71]
ZIF/PAN	In situ growth on PAN nanofibers	Leaf-like bimetallic ZIF	Hierarchical morphology; immobilized, recoverable	Norfloxacin degradation	[75]
NH_2_-MIL-88B(Fe)/PVDF	Non-solvent-induced phase separation	NH_2_-MIL-88B(Fe)	Fe photo-Fenton catalyst; hydrophilic filler; PMS activation	Tetracycline removal	[76]
Co-MOF@PDA/PVDF	Solvothermal (waste-PET linker) + PDA cross-linking + vacuum filtration	Co-MOF (PET-derived)	Co-MOF nanorods anchored on PVDF	Antibiotic degradation (visible light/PMS)	[77]
PVA/GO@MOF	Suction filtration cross-linking	UiO-66-NH_2_	Underwater superoleophobic; photothermal + photocatalytic self-cleaning	Oil/water + foulant removal	[36]
TFN-NC3 (NiFe-MOF/CQD)	Interfacial polymerization	NiFe-MOF	CQD-modified MOF filler in polyamide layer	Self-cleaning nanofiltration	[78]
CB/Cu-MOF/STA	In situ growth + stearic acid	Cu-MOF	Superhydrophobic; anti-biofouling	Oil/water + dye degradation	[79]
UiO-66-NH_2_(Zr/Hf)/α-Al_2_O_3_	Reaction seeding	UiO-66-NH_2_	Chemically & aqueous stable film	Cr(VI) reduction	[31]
MOF-199/Ni foam	Electrodeposition	MOF-199 (Cu)	50 nm particles; unsaturated Cu sites	H_2_ production	[30]
Pt@UNPM/Pt	In situ growth on polymer substrate	Zr-based, defect-rich, Pt-loaded	Lattice defects; short electron transfer	H_2_ production	[37]
Pt@UCM/CdS	In situ growth + liquid-phase sulfidation	Pt-doped UiO-66-NH_2_	3D interpenetrating S-scheme network; photocorrosion-buffered	H_2_ production	[80]
NH_2_-MIL-125(Ti)/PVDF	Solution casting (1–10 wt%)	Cu-modified NH_2_-MIL-125(Ti)	Polymer-protected unstable MOF	H_2_ production	[81]
(**B**)					
Membrane	Reaction	Light/medium	Key performance metrics	Stability	Ref.
SA/MOF (Ir_1_)	CO_2_RR	Visible, gas–solid	HCOOH near-100% selectivity; AQE 15.76% at 420 nm	Not reported	[34]
SA/MOF (Pd_1_)	O_2_→H_2_O_2_	Visible, gas–solid	H_2_O_2_ 10.4 mmol·g^−1^_cat·h^−1^	Not reported	[34]
Ni_3_HITP_2_/rGO	CO_2_RR	Visible light	CO 3.8 × 10^4^ μmol·h^−1^·g^−1^; selectivity 91.74%	Multiple cycles (recoverable film)	[32]
NMD/PAN	CO_2_RR	Visible light	CO 1641 μmol·g^−1^·h^−1^; 91% selectivity	71% activity after 7 cycles	[70]
[DMC@cMOF]-PVK	CO_2_RR	Visible light, water donor	CO 133.36 μmol·g^−1^·h^−1^; 8× areal gain (10-layer)	Not reported	[71]
NH_2_-MIL-88B(Fe)/PVDF	PMS-assisted degradation	PMS activation	High-efficiency tetracycline removal	Not reported	[76]
Co-MOF@PDA/PVDF	Tetracycline degradation (PMS)	Visible light + PMS	97.63% in 25 min (k = 0.1645 min^−1^)	>85% after 5 cycles	[77]
PVA/GO@MOF	Emulsion + self-cleaning	Sunlight/photothermal	>99% emulsion separation; 99.9% foulant removal in 50 min	20 cycles, flux > 1250 L·m^−2^·h^−1^·bar^−1^	[36]
TFN-NC3	Self-cleaning nanofiltration	Visible light	20.2 L·m^−2^·h^−1^·bar^−1^; 98.2% Na_2_SO_4_; >86% dyes; >93.4% tetracycline rejection	Flux recovery > 95% over 5 cycles	[78]
UiO-66-NH_2_(Zr/Hf)	Cr(VI) reduction	Simulated & natural sunlight	High-efficiency Cr(VI) reduction	>94% after 20 cycles	[31]
MOF-199/Ni	H_2_ evolution	Visible light	High H_2_ production rate; reusable	Reusable, no significant loss	[30]
Pt@UCM/CdS	H_2_ evolution	Pure water/seawater	47.45 mmol·m^−2^·h^−1^	1100 h pure water (75% recoverable); 600 h seawater	[80]
NH_2_-MIL-125(Ti)/PVDF	H_2_ evolution	Aqueous/acetonitrile	389.1/466.0 μmol·h^−1^·m^−2^ (10 wt%)	20 h, crystalline integrity retained	[81]

**Table 3:** (**A**) Fabrication and structural features of thermocatalytic MOF membranes. (**B**) Catalytic performance and testing conditions of thermocatalytic MOF membranes.

(**A**)					
Membrane/Catalyst	Fabrication route	MOF type	Key structural features	Catalytic application	Ref.
ZIF-8@CFF	Coating on carbon fiber fabric	ZIF-8 (Zn)	Uniform NP coating; temperature-gradient-driven Zn^2+^ catalysis	Antibacterial (H_2_O_2_ generation)	[40]
Cu-BTC/PIL	In situ growth on poly(ionic liquid) fibers	Cu-BTC (HKUST-1)	Sustained Cu^2+^ + ROS release; multifunctional wound dressing	Antibacterial	[38]
UiO-66/PVDF (3 routes)	Blending/one-step/modulated	UiO-66	Route-dependent encapsulation, defect density (Cl/Zr 0.04–0.10), crystallite exposure	Oxidative desulfurization	[39]
(**B**)					
Membrane	Reaction	Testing conditions	Key performance metrics	Stability	Ref.
ZIF-8@CFF	Antibacterial	5–10 °C temperature gradient	96% inactivation (*E. coli*, *S. aureus*)	30 days, no detachment	[40]
UiO-66/PVDF (blending)	Desulfurization	Model fuel	54% in 2 h (MOF encapsulated)	—	[39]
UiO-66/PVDF (one-step)	Desulfurization	Model fuel	96% in 2 h (Cl/Zr = 0.10)	No leaching, 5 reuse cycles	[39]
UiO-66/PVDF (modulated)	Desulfurization	Model fuel	69% in 2 h (Cl/Zr = 0.04)	—	[39]

**Table 4:** Predicted thermally stable MOF families, stability windows and prospective high-temperature industrial membrane-catalysis reactions.

MOF Family	Approx. Thermal Stability (Inert)	Target Industrial Reactions	Expected Membrane Function	Major Remaining Challenges
UiO-66/UiO-67 (Zr-based)	300–420 °C [91]	CO_2_ methanation; selective VOC oxidation	Catalytic layer + in situ water removal by permeation	Hydrothermal stability under mixed-gas atmosphere; defect evolution
MIL-53(Al, Cr)	250–380 °C [92]	Light-alkane dehydrogenation	Reactant enrichment; selective product permeation	Membrane brittleness under thermal cycling
ZIF-8	250–320 °C	Methanol synthesis from CO_2_/CO	Molecular sieving for product extraction	Ligand decomposition under humid feed gas

**Table 5:** Recommended minimal reporting guidelines for catalytic MOF-membrane studies.

Category	Mandatory Reporting Parameters
General material information	Full MOF chemical formula; membrane fabrication route; membrane thickness; active-phase loading mass; phase-purity evidence (PXRD); cross-section morphology
Electrocatalysis	Normalization basis (geometric area/catalyst mass); cell configuration; electrolyte composition; applied potential/current density; Faradaic efficiency; absolute product yield; long-term galvanostatic stability
Photocatalysis	Light-source type, exact irradiance; reactor geometry; reaction medium; performance normalization basis; absolute product formation rate; apparent quantum efficiency (AQE); multi-cycle recycling data
Thermocatalysis	Reaction temperature, pressure, feed-gas composition; space velocity; conversion and selectivity; time-on-stream stability; metal-leaching test results
Cross-cutting requirement	Clear statement whether the material is an intact MOF membrane or MOF-template-derived catalyst

## Data Availability

No new data were created or analyzed in this study. Data sharing is not applicable to this article.
